# A Colorimetric Multimetabolite Assay for Quantitative Measurement of Keto Acids in Urine for At‐Home Monitoring of Metabolic Disorders

**DOI:** 10.1155/jamc/4116313

**Published:** 2026-02-27

**Authors:** Dipanjan Bhattacharyya, Abby Kropielnicki, Brian L. Lee, Yeganeh Khaniani, Marcia A. LeVatte, David S. Wishart

**Affiliations:** ^1^ Department of Biological Sciences, University of Alberta, Edmonton, Alberta, T6G 2E9, Canada, ualberta.ca; ^2^ Department of Computing Science, University of Alberta, Edmonton, Alberta, T6G 2E8, Canada, ualberta.ca; ^3^ Department of Laboratory Medicine and Pathology, University of Alberta, Edmonton, Alberta, T6G 2B7, Canada, ualberta.ca; ^4^ Faculty of Pharmacy and Pharmaceutical Sciences, University of Alberta, Edmonton, Alberta, T6G 2H7, Canada, ualberta.ca

**Keywords:** 24-dinitrophenylhydrazine, colorimetric assay, keto acid, maple syrup urine disease, nuclear magnetic resonance spectroscopy, phenylketonuria, urine

## Abstract

Inborn errors of metabolism such as phenylketonuria (PKU) and maple syrup urine disease (MSUD) can cause severe developmental problems. Both conditions can lead to harmful levels of keto acids in biofluids—phenylpyruvic acid (PPA) in PKU and branched‐chain α‐keto acids in MSUD. Monitoring urinary keto acids helps track dietary adherence and reduces the risk of metabolic crisis. However, current at‐home tests are qualitative and difficult to interpret, while existing metabolomic assays require expensive equipment and must be conducted in a lab. This study aimed to develop a simple, quantitative, rapid, at‐home assay for detecting multiple urinary keto acids associated with PKU and MSUD. A modified two‐step 2,4‐dinitrophenylhydrazine (DNPH)–based multimetabolite assay was developed, where sodium hydroxide (NaOH) converts the yellow hydrazone precipitate to a stable amber solution, enabling quantification of multiple keto acids (700–7200 μM) within 10 min. The assay was validated using spiked urine samples and adapted into a prototype at‐home kit using caprolactam‐immobilized NaOH. Nuclear magnetic resonance (NMR)–based metabolomics was used as a reference method to authenticate readings from a PKU patient. Correlation studies demonstrated strong linearity for MSUD (*R* ^2^ = 0.91–0.96)‐ and PKU (*R* ^2^ = 0.95–0.99)‐spiked samples. Quantification of keto acids in authentic PKU urine samples showed excellent agreement with the results of quantitative NMR‐based metabolomics assays (*R* ^2^ = 0.99). Low‐cost, at‐home colorimetric tests for urinary keto acids could enable screening, detection, and monitoring of PKU and MSUD in the 90% of the world without access to advanced metabolic clinics.

## 1. Introduction

Both phenylketonuria (PKU) and maple syrup urine disease (MSUD) are inborn errors of metabolism (IEMs) that impair the body’s ability to break down specific amino acids. Both conditions require lifelong dietary management and regular monitoring to prevent serious health complications. Regular testing for both conditions is especially important for infants and children, as it helps ensure metabolic control, prevents the accumulation of toxic by‐products, and supports normal growth and neurological development. Close monitoring allows for timely dietary adjustments and helps maintain safe amino acid levels, which is critical for long‐term health and quality of life. Monitoring patients with PKU and MSUD can be challenging due to the need for strict dietary adherence, frequent blood tests, and timely access to specialized lab or metabolomic services. Families often face a significant burden to manage the complex care routines, and variability in monitoring protocols across clinics can result in inconsistent treatment. Faster, simpler, more consistent, less‐invasive, at‐home tests would reduce this burden. However, to develop these kinds of at‐home metabolomic tests, it is important to understand the biochemistry of both disorders and briefly review past attempts at developing at‐home tests.

For MSUD, defects in the branched‐chain α‐keto acid dehydrogenase complex (BCKD) prevent the breakdown of three essential branched‐chain amino acids (BCAAs), leucine, isoleucine, and valine (see Scheme S1) [1]. As a result, these BCAAs and their α‐keto acids α‐ketoisocaproic acid (KIC), α‐ketoisovaleric acid (KIV), and α‐keto‐β‐methylvaleric acid (KMV) accumulate in the brain (10–20X higher), blood (30X greater), and urine (40–8000‐fold higher) [2–4]. As these α‐keto acids are neurotoxins, their accumulation can cause brain damage, hallucinations, seizures, and even coma [2–4].

PKU results from reduced phenylalanine hydroxylase (PAH) activity, causing toxic levels of phenylalanine and its α‐keto acid phenylpyruvic acid (PPA) [5] to accumulate in the blood (and brain) of PKU newborns. Blood phenylalanine levels can increase up to 20X higher than normal [5], while urinary PPA levels can increase to 10,000X higher than normal [6, 7]. Left unchecked, excess phenylalanine in the brain can lead to microcephaly, severe brain damage, mental retardation, and seizures in PKU patients [4, 5].

Both MSUD and PKU patients are discovered via newborn screening of dried blood spots (DBSs) within two to 3 days of birth. For MSUD, the DBS findings are further confirmed through additional testing for BCAAs in plasma using quantitative liquid chromatography‐tandem mass spectrometry (LC‐MS/MS) metabolomic analysis and testing for urinary keto acids by gas chromatography MS (GC‐MS) analysis [8]. Alternately, testing can be done by using the nonquantitative dinitrophenylhydrazine (DNPH) [9] chemical assay if GC‐MS is unavailable. After diagnosis, MSUD management requires strict restriction of dietary BCAAs with regular monitoring of urinary α‐keto acid levels using DNPH‐based spot tests in either out‐patient settings or at‐home [8].

PKU can be diagnosed via the detection of elevated phenylalanine levels in newborn DBS samples. After diagnosis, PKU patients must have their blood levels of phenylalanine and phenylketones, such as PPA, measured weekly and diets closely monitored. However, testing often falls off with age. As PKU patients reach adolescence, their desire to maintain their restrictive diet wanes [10]. Because metabolic decompensation can occur in both PKU and MSUD, especially during illness, fasting, or dietary lapses, quick, simple, noninvasive, at‐home tests would substantially improve day‐to‐day monitoring for families.

There are a few qualitative colorimetric bioassays to monitor α‐keto acids. For MSUD patients, the DNPH assay (also known as Brady’s test) is used by parents to test for urinary α‐keto acids in their children. The reagent is mixed one to one with urine and parents look for a yellow–white precipitate (hydrazones) after 10 min [11]. The results are scored from 0 (clear) to 4+ (white–yellow precipitate) to determine whether keto acids are present (+) or absent (0 or clear). However, the results are difficult to interpret as there is little differentiation between 1+ and 4+ results, providing limited information for the parents. The DNPH assay has also been shown to detect keto acids that buildup in urine from other IEMs, including PPA in PKU [9]. For PKU patients, the ferric chloride (FeCl_3_) test, incorporated into Phenistix tests [12], has been employed to detect the α‐keto acid, PPA, in urine [9]. When the reagent is added appropriately to urine, it produces a blue–green color. However, this qualitative test is difficult to conduct, often misses the condition in its early stages, fails to detect mild cases, and does not provide a quantitative readout of PPA levels. Overall, currently available qualitative colorimetric assays lack the reliability and resolution needed for routine at‐home monitoring.

While current standards‐of‐care for PKU and MSUD in the developed world rely on plasma‐based or DBS biomarkers measured via LC‐MS/MS methods, it is important to remember that only 18% of newborns born today have access to LC‐MS/MS‐based screening and very few IEM patients have ready access to metabolic clinics that can perform routine LC‐MS/MS blood‐based monitoring [13–16]. Therefore, for the vast majority of PKU or MSUD patients living today and for the vast majority of PKU or MSUD newborns born today, the only option for detecting, screening, or monitoring PKU or MSUD would be some kind of simple colorimetric test [17]. Even in wealthier countries, with current clinical practice, turnaround time for DBS results can be five to 6 days, delaying assessment during metabolic crises. In such crises, when plasma levels of phenylalanine for PKU or BCAA for MSUD rise above established threshold levels, their respective α‐keto acids also rise linearly in plasma [18, 19]. Once plasma α‐keto acids exceed renal thresholds, they are excreted in high levels in urine [18, 19] and can be readily detected. Notably, in our study, urinary PPA was detectable in a well‐controlled PKU patient who was not in metabolic crisis, suggesting that α‐keto acids may be measurable in urine even at subthreshold plasma concentrations. This expands the potential utility of urinary keto acid detection beyond acute settings and supports its use in routine, noninvasive monitoring as recently suggested by Wild et al. [20]. Given these facts, our aim was to develop a low‐cost, noninvasive, simple, quantitative, reliable, at‐home, or in‐clinic colorimetric assay that could be used at home or in remote, low‐resource settings with limited access to LC‐MS/MS–equipped laboratories.

To address the limitations of existing bioassays, we developed a simple and inexpensive colorimetric multimetabolite assay for the rapid, quantitative detection of multiple α‐keto acids in urine using DNPH. Our assay uses a DNPH‐based colorimetric reaction that forms amber hydrazones upon alkalinization, enabling direct measurement of urinary α‐keto acids. While similar DNPH‐based reactions had been tested years ago, they required acid‐treated urine or acid‐precipitated blood followed by solvent extraction before reaction with a base [21, 22]. Direct detection using DNPH and NaOH was previously only successful with pure solutions that had to be heated at high temperatures for extended periods [23, 24]. Our optimized assay eliminates these constraints, working directly with urine samples of varying hydration levels to provide consistent, reproducible results. It enables the detection of α‐keto acids including KIC, KIV, and KMV, expected to be elevated in urine from MSUD patients as well as PPA, which is typically elevated in PKU [9, 23]. Additionally, we adapted this multimetabolite assay into a simple, at‐home kit, which could facilitate the rapid detection and monitoring of these α‐keto acids in MSUD and PKU patients. The methods used to optimally detect and quantify these keto acids in spiked human urine and patient urine samples, as well as the successful development of a prototype at‐home kit, are described below.

## 2. Materials and Methods

### 2.1. Materials and Other Consumables

Information about the reagents and consumables used for the DNPH assays and nuclear magnetic resonance (NMR) spectroscopy, and consumables purchased for the at‐home assay are provided in the supporting information.

### 2.2. Urine Samples

Urine samples were obtained via informed consent and conducted in compliance with the ethical standards of, and approved by, the University of Alberta’s Human Research Ethics Board (HREB) biomedical ethics committee (HREB #Pro00092437, June 3, 2024). Mid‐stream urine samples were collected from healthy Canadian volunteers of diverse ages, dietary preferences, and ethnic backgrounds (60% males, aged 22–41 years, mean 35.4 years of 10% African, 20% each of Asian–Chinese, Caucasian, and Middle Eastern, and 30% East Indian ethnicity [25]). Based on various features (color, creatinine levels, etc.), these urine samples also exhibited a broad range of hydration levels. A local pooled urine (L‐PU) was created from these samples as previously described [26]. Commercial pooled urine (C‐PU; 12 donors; 2 L), purchased from Lee BioSolutions (Maryland Heights, USA), was prepared as previously described [27]. Four different PKU urine samples from an adult patient, adhering to a low‐phenylalanine diet, were obtained after an overnight fast (before breakfast), after breakfast, after dinner, and after lunch. Urine samples were immediately aliquoted into 1.5‐mL microfuge tubes and frozen at −80°C until used. Prior to analysis, samples were thawed immediately and then centrifuged to pellet any precipitated material. Cloudy samples, when present, were passed through a 0.22‐μm sterile filter. Details about the storage of these samples are provided in Table S1.

### 2.3. Principle of the DNPH Plus NaOH Assay

**FIGURE 1:**
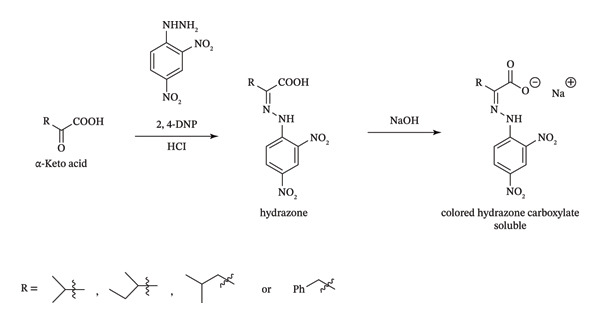
Formation of colored hydrazone carboxylate from α‐keto acids. Under acidic conditions, the ketone functional group of the urinary α‐keto acid reacts with the hydrazine functionality of DNPH (dinitrophenylhydrazine) to produce a hydrazone derivative, which, upon treatment with NaOH, becomes a highly conjugated hydrazone carboxylate, which is reddish‐brown or amber in color. Abbreviations: 2,4‐DNP: dinitrophenylhydrazine; HCl: hydrochloric acid; NaOH: sodium hydroxide.

This method relies on the well‐established reaction between DNPH and α‐keto acids, which occurs under acidic conditions. The ketone functional group of the urinary α‐keto acid reacts with the hydrazine functionality of DNPH to produce a hydrazone derivative. Upon treatment with NaOH, these hydrazones become highly conjugated, and in turn, the solution becomes amber–brown in color (Figure 1) with absorbance maxima at 430 nm (A_430_) and 530 nm (A_530_). The following sections describe the optimized conditions that enable the reaction to proceed directly in untreated urine without heating, precipitation, or extraction.

### 2.4. Pyruvic Acid “Testing” Assay

Pyruvic acid is an α‐keto acid that is found at low levels in all human urine samples. To test the sensitivity of the proposed colorimetric multimetabolite assay and to optimize all the assay parameters, an inexpensive testing reagent, pyruvic acid, was chosen. Therefore, a pyruvic acid “testing” assay was developed. For this assay, 50 μL of the individual diluted urine samples, with or without added pyruvic acid, was aliquoted. Then, 10 μL of the 4.44 mM DNPH solution (8.8 mg DNPH/10 mL 2 M hydrochloric acid) was added, mixing thoroughly by vortexing and incubated at RT for 10 min under quiescent conditions. Then, 50 μL of the 6 M NaOH solution was added and mixed thoroughly by vortexing. Finally, 100 μL of the reaction mixture was transferred into individual wells of a 96‐well plate and the absorbance values at 430 nm (A_430_) and 530 nm (A_530_) (BioTek Synergy HT UV–vis spectrophotometer) were measured exactly 2 min later.

### 2.5. Keto Acids and PPA Assays

To develop the keto assay of interest, 50 μL of the individual diluted urine samples or L‐PU (with or without added α‐keto acids or PPA) was tested as described for pyruvate, except 30 μL of 4.44 mM DNPH was added and incubated at RT for 5 min. When NaOH solution was added, the resultant solution was mixed by pipetting up and down using a multichannel pipettor rather than vortexing.

### 2.6. Preparation of the Pyruvate‐, Keto Acid–, or PPA‐Spiked Urine Samples

Keto acid–spiked urine samples were prepared using six different α‐keto acids: pyruvate, KIC, KIV, KMV, PPA, and α‐ketoglutarate (AKG). Urine samples were spiked with pyruvate to optimize the DHPH + NaOH assay using an inexpensive keto acid (as mentioned above), with the keto acids, KIC, KIV, and KMV, to mimic urine samples from MSUD patients and with PPA to mimic urine samples from PPU patients. Additional MSUD and PKU samples were also spiked with pyruvate and AKG, which are endogenous α‐keto acids found in nearly all normal urine samples. Methods detailing how these spiked urine samples were prepared from 10 mM stock solutions are found in the supporting information.

### 2.7. Randomly Spiked Branched Chain α‐Keto Acids or PPA Samples

To prepare 35 urine samples spiked with 300–1600 μM total branched‐chain α‐keto acids or PPA, urine samples that were 3X and 4X diluted, respectively, were randomly spiked with different amounts of 10 mM branched‐chain α‐keto acids or 10 mM PPA, respectively. These spiked urine samples were then assayed for the presence of these α‐keto acids using our modified keto acid/PPA DNPH assay.

### 2.8. Preparation of PPA and Branched‐Chain α‐Keto Acid Calibration Curves

Details about generation of the 0–1600 μM PPA or MSUD α‐keto acid calibration curves using the 4X or 3X diluted L‐PU sample, as well as details about background subtraction and calculations of standard deviations (SD), coefficients of variance (CV), and coefficient of determination (*R* ^2^), LODs (3 × SD/slope), and LOQs (10 × SD/slope) [28], are provided in the supporting information.

### 2.9. Preparation of PPA and Keto Acid Correlation Curves From Spiked Samples

Details about how the correlation curves for the 4X diluted, variably spiked PPA urine samples or the 3X diluted, variably spiked branched‐chain α‐keto acid urine samples were generated are provided in the supporting information.

### 2.10. Components of the MSUD/PKU At‐Home Assay

The prototype at‐home MSUD/PKU multimetabolite testing kit includes assay vials (marked with one red line) containing a capped, acidic solution of premeasured DNPH (500 μL, 10 mM in 2M HCl), a bottle of distilled Milli‐Q water containing a dropper, urine sample cups, gloves, NaOH stopper caps, benzalkonium chloride (BZK) antiseptic wipes, a 3D‐printed vial stand with a printed color chart sticker for reference to keto acid levels, and an instruction manual. The model of the 3D‐printed vial stand (80 × 50 × 82 mm) was designed with Autodesk Fusion 360 software (California, United States) and 3D‐printed using Elegoo rapid polyethylene terephthalate glycol–modified (PETG) filament on a Bambu Lab P1S 3D Printer (Shenzhen, China). It is important to note that this prototype kit was designed and used for research purposes only.

### 2.11. Preparation of Caprolactam–NaOH‐Containing Caps

To prepare the plastic stopper caps (7.2 mm diameter × 10.2 mm length) containing NaOH, solid caprolactam was heated in a glass vial to 70°C in a water bath until fully melted. NaOH pellets were carefully ground into a fine powder using a mortar and pestle to ensure uniformity and facilitate dissolution. Next, 70 μL of melted caprolactam was rapidly pipetted onto preweighed NaOH (125 mg) inside each cap. The caps were then cooled at RT for 10 min and sealed with parafilm and stored in a desiccator.

### 2.12. Preparation of α‐Keto Acids and PPA Standard Solutions for Standard Color Charts

The 0, 500, 1000, 2000, and 4000 μM PKU standards or 0, 375, 750, 1500, and 3000 μM MSUD calibrants were prepared in C‐PU from the 8000 μM PKU stock solution or the 6000 μM MSUD stock solution, respectively. The MSUD 6000 μM stock solution contained 2000 μM each of KIC, KIV, and KMV. The 8000 μM PKU stock solution included the keto acid PPA, L‐phenylalanine, glycine, and mandelic acid as they were significantly elevated in PKU urine [29], mimicking the composition of patient samples. To ensure a physiologically relevant AKG concentration, 200 μM AKG was spiked into all MSUD or PKU calibrants yielding final keto acid concentrations of 0, 575, 950, 1700, 3200, and 6200 μM for the MSUD standards and 0, 700, 1200, 2200, 4200, and 7200 μM for the PKU standards. Details of preparation of the MSUD and PKU standards are provided in the supporting information.

### 2.13. MSUD or PKU Colorimetric Kit Color Chart Generation

To create a color chart and establish a linear color range, we determined which dilutions of MSUD or PKU keto acids produced visually discernible colors. C‐PU was spiked with MSUD keto acids (KIC, KIV, KMV, AKG, and pyruvate) at final concentrations of 0, 1600, 3600, 6000 μM or with PKU keto acids (PPA plus AKG) at 0, 2400, 4400, 6400 μM. These spiked samples were diluted 3X, 4X, 6X, 10X, and 20X with water and reacted using the at‐home assay. To minimize oversaturation at lower dilutions, the 3X and 4X dilutions used 4.44 mM DNPH, while the 6X, 10X, and 20X dilutions used 10 mM DNPH. After the reaction, 200 μL of the colored solutions was transferred to a 96‐well plate for spectrophotometric analysis. A_530_ from the MSUD keto acids and the A_430_ of the PKU keto acids were plotted against the spiked concentrations. Linear calibration curves and *R* ^2^ were calculated using Microsoft Excel. The LOD and LOQ were also determined from a baseline blank prepared using Milli‐Q water in the place of the C‐PU sample. Values were calculated using the same method described in Subsection 2.8.

To prepare the color chart, PKU standards (0, 700, 1200, 2200, 4200, and 7200 μM) and MSUD standards (0, 575, 950, 1700, 3200, and 6200 μM) prepared in 1 mL (described in the above section) were reacted via the prototype at‐home assay (see section below) and photographed using a Pixel 9 Pro cell phone. The resulting colors of the PKU and MSUD standards in the colored image were captured using the Canva (https:/canva.com) eye dropper tool, and the HEX values were used to create colored squares for each chart.

### 2.14. Prototype At‐Home Kit Assay

The prototype at‐home assay uses 7‐mL vials, premarked with a red line, to facilitate a 20X urine dilution. Each vial is preloaded with 500 μL acidic 10 mM DNPH (198 mg DNPH/100 mL 2M HCl). For both the MSUD and PKU assays, the patient adds Milli‐Q water (4750 μL) to the red line on the vial using the provided water bottle. Using the provided eye dropper (marked at 0.25 mL for easy measurement), they transfer 0.25 mL (250 μL) of urine sample from the urine collection cup to the vial, achieving the 20X dilution for urine (250 μL urine in 4750 μL Milli‐Q water). After adding the urine, the patient places the vial in the 3D printed stand and starts a timer. After 5 min, they remove the black twist cap and insert the caprolactam–NaOH stopper cap into the vial (filled with 125 mg NaOH, 70 μL caprolactam). The black twist cap is then secured back onto the vial. The vial is then inverted repeatedly for 5 min to ensure complete dissolution of the NaOH in the stopper cap. Finally, the patient places the reaction vial back in the stand and compares the color to the provided reference chart.

### 2.15. Stability of the Caprolactam–NaOH Caps

To determine the stability of the caprolactam–NaOH caps, 70 μL of melted caprolactam was rapidly pipetted onto 125 mg of solid NaOH in four caps and stored for 1, 30, and 60 days at RT (covered by parafilm). Unspiked (0 μM) and spiked (1500 μM, containing 500 μM each of KIC, KIV, and KMV) C‐PU samples were prepared freshly and assayed on Days 1, 30, and 60 by the at‐home assay. Both freshly prepared caprolactam–NaOH caps and those stored at RT were assayed on the indicated days after 5 min of reaction of the DNPH with the spiked and unspiked C‐PU samples.

### 2.16. Stability of the DNPH Solution

To test the stability of the DHPH reaction vials, premeasured 10 mM DNPH (in 2 M HCl) was aliquoted into 50 reaction vials and stored at 4°C or at room temperature (21°C, in the dark) for 0, 1, 2, 3, 4, 8, and 12 weeks. Dried NaOH stoppers/caps were prepared as described above (see Subsection 2.11) and stored and covered with parafilm in a desiccator until used. Unspiked (0 μM) and 4000 μM PPA‐spiked C‐PU samples were prepared, aliquoted into 1.5‐mL Eppendorf tubes to avoid freeze–thaw cycles, and stored at −80°C until tested to ensure the stability and consistency of the samples over the 12 weeks of testing. At the indicated time points, DNPH vials (stored at RT or 4°C) and spiked urine samples (stored at −80°C) were removed, thawed, and reacted via the at‐home assay. Absorbance at 430 nm (A_430_) was measured after 5 min. Absorbances of the 0 and 4000 μM PPA‐spiked C‐PU reactions over the 12 weeks was assessed as signal‐to‐noise (S/N) ratios as defined in the following equation:

(1) SN=A430 of 4000 µMA430 of 0 µM.

### 2.17. Identification of Keto Acids Interfering With DNPH + NaOH Reaction

To determine the extent of reaction of abundant metabolites in normal or PKU or MSUD urine with DNPH + NaOH, 0, 20, 100, 300, and 500 μM acetaldehyde prepared in water, or 0, 100, 600, and 1200 μM acetone, prepared in water, was reacted via the at‐home assay. For oxaloacetic acid (OAA), phenylacetic acid, pyruvate, AKG, 4‐hydroxyphenylphruvic acid (4‐HPPA), and PPA, solutions were prepared in C‐PU and quantified by the at‐home assay. After 5 min, absorbance at 530 nm was measured and plotted against the spiked‐in concentrations of acetone and acetaldehyde. For OAA, PPA, pyruvate, AKG, 4‐HPPA, and PPA, the absorbance of the unspiked C‐PU sample was subtracted from these reaction absorbances. These net absorbances of the individual metabolites were scored as a percentage of the absorbance of PPA and then multiplied by 100 (as shown in the following equation:

(2) % PPA absorbance=absorbance of metaboliteabsorbance of PPA ×100.

### 2.18. Quantification of Keto Acids and Other Metabolites by NMR Metabolomics

C‐PU and PPA clinical urine samples were analyzed for various metabolites including 3‐phenyllactate, 3‐indoleacetic acid, acetate, acetone, acetaldehyde (both aldehyde and hydrate forms), creatinine, glycine, hippurate, mandelate, OAA, phenylacetic acid, phenylalanine, phenylglyoxylate, PPA, pyruvate, AKG, *o*‐hydroxyphenylacetic acid, and 4‐hydroxyphenylpyruvic acid (4‐HPAA) using previously described NMR methods [26]. The peak positions for OAA and 4‐HPPA were difficult to identify in the initial analysis, necessitating their addition to PKU clinical samples for confirmation. To determine their precise peak positions, OAA and 4‐HPPA were individually spiked into the four PKU clinical samples and compared to a PPA sample spiked with an equivalent amount of water. For each PPA clinical sample, three conditions were prepared: (1) spiked with water (control); (2) spiked with 200 μM 4‐HPPA; and (3) spiked with 200 μM OAA. Additionally, for PPA standards (0–7000 μM PPA + AKG) prepared in C‐PU, PPA and AKG concentrations were quantified by NMR. Aliquots of C‐PU, the PPA standards, the 4‐HPPA– or OAA‐spiked samples, and the four PKU urine samples were mixed with 5 × NMR buffer, vortexed, and centrifuged, and then, the supernatant was loaded into 3‐mm NMR tubes.

NMR spectra were recorded on a 700‐MHz Avance III HD Bruker (Bruker Biospin, Rheinstetten, Germany) NMR spectrometer equipped with a cryogenically cooled triple resonance probe (TCI) as previously described [26, 27]. All spectra were processed using the TopSpin software package Version 3.5 pl.7 (Bruker Biospin, Rheinstetten, Germany). The Chenomx NMR Suite software Version 8.3 (Chenomx, Inc., Alberta, Canada) was used to identify and quantify the metabolites in the urine samples. Each metabolite concentration was corrected for dilution with the 5 × NMR buffer by dividing by 0.8. The μM/mM creatinine was calculated by dividing the μM of metabolite by the μM creatinine and then multiplying by 1000 μM/mM. Additional details about sample preparation, NMR spectra acquisition, and quantification are provided in the supporting information.

### 2.19. Preparation of NMR and PKU Clinical Urine Sample Correlation Curve and Bland–Altman Analysis

The concentrations of the 0–7000 μM PPA + AKG calibrants, determined by NMR, were plotted against the A_530_ of the 20X diluted calibrants, measured via the at‐home assay, to generate a linear calibration curve. The concentrations of the keto acids in the four PKU urine samples were extrapolated from this NMR linear calibration curve. The assay‐determined concentrations were then plotted against the summed NMR‐determined concentrations of keto acids in each sample—PPA, AKG, 4‐HPPA, OAA, pyruvate, and phenylacetic acid—which are elevated in PKU patients [29] and react in our assay. This plot generated a correlation curve for the PKU urine samples. *R* ^2^ was calculated using Microsoft Excel. Bland–Altman analysis was performed on the means and differences between the NMR‐ and assay‐determined concentrations (with and without creatinine normalization). Additionally, the means, differences, and SD of differences between the NMR values and the colorimetric assay‐determined concentrations (with and without creatinine normalization) were also calculated using Microsoft Excel.

## 3. Results

### 3.1. Assay Optimization Using Pyruvic Acid, MSUD α‐Keto Acids, PPA, and Endogenous Keto Acids

The results in this section have been abbreviated. See the supporting information for more details. Pure, commercially available pyruvic acid was reacted with DNPH and NaOH to produce a colored product detectable at 416 nm [23]. However, this reaction was not previously reported with urine. We demonstrated that pyruvic acid, a low‐abundance urinary metabolite (normal range 7–32 μM/mM creatinine [4, 30]), could be detected in urine using DNPH (10 μL, 4.44 mM DNPH) and NaOH (50 μL, 6 M). With increasing concentrations of pyruvic acid, a distinct color gradient of reddish‐amber products was observed, two absorbance maxima were detected (430 nm and 520 nm, Figure S1), and a linear response to increasing pyruvic acid was seen (data not shown). While normal urine samples yielded similar reactions, intensely yellow‐colored urine samples yielded inconsistent results, suggesting that further optimization was needed.

For MSUD, urinary α‐keto acids ranging from 300 μM to 2000 μM are desirable and reflect BCAA plasma levels in a healthy range of 75–300 μM for leucine and 200–400 μM each for isoleucine and valine [4, 11, 18, 31, 32]. We spiked urine with 0–2000 μM pyruvate and then added increasing amounts of the DNPH (10, 15, 20, and 30 μL) with 30 μL yielding the best color gradient. For all subsequent assays, when 50 μL of urine was tested, 30 μL of the DNPH solution was added, followed by 50 μL of 6 M NaOH. However, some precipitates formed when the highest concentration (2000 μM) of pyruvate was reacted. We used simple dilution to minimize the possible effects of interfering compounds and to avoid the precipitation of the hydrazone compounds in patient samples containing high amounts of α‐keto acids. With 3X diluted urine, consistent colors were observed, and no precipitation was seen.

After optimizing on pyruvic acid, we then demonstrated that the assay worked with a single α‐keto acid, KIC (500–4000 μM), spiked into 3X diluted L‐PU. With DNPH added, a yellow reaction mixture containing increasing amounts of yellowish‐white precipitate was observed (Figure S2a). When NaOH was then added, the yellowish‐white precipitates disappeared and reddish‐amber to dark amber products were seen (Figure S2b), demonstrating that the assay works well with an α‐keto acid found in urine of MSUD patients.

While three α‐keto acids (KIC, KIV, KMV) accumulate to high levels in the urine of MSUD patients, other less abundant and “normal” α‐keto acids such as pyruvate (7–32 μM/mM creatinine [4, 33]) and AKG (4–52 μM/mM creatinine [4, 33]) are also present in urine (about 900 μM when 10 mM creatinine is present). When 3X diluted normal urine sample was spiked with total 300 μM normal α‐keto acids (100 μM pyruvate and 200 μM AKG) and up to 1500 μM MSUD α‐keto acids (KIV, KMV, and KIC; see Table S2 for amounts of each), creating 300–1800 μM total α‐keto acids and then assayed using our DNPH protocol, amber–brown products, whose color increased in the intensity with increasing amounts of α‐keto acids, were observed (Figure S3a). Absorbance maxima for the α‐keto acids varied slightly (Figure S1) with A_430_ and A_530_ selected where the absorbances were maximal for most of these keto acids. With increasing α‐keto acids, a linear response curve was generated at A_430_ and A_530_ with almost identical *R* ^2^ (0.99; Figure S3b), demonstrating that our modified DNPH assay can quantitatively measure 300–1800 μM total α‐keto acids in 3X diluted urine. Furthermore, it showed that the test would not confuse even extreme “normal” samples (containing high‐normal levels of common α‐keto acids such as pyruvate and AKG) with MSUD or with an MSUD “α‐keto acid flare‐up.”

Phenylpyruvate concentrations in the urine of PKU patients can be extremely high, up to 31 mM/mM creatinine [4]. This is more than the concentrations expected for urinary branched‐chain α‐keto acids in MSUD patients. Because we observed the precipitation of the colored carboxylate product at 2000 μM pyruvic acid, we modified our PKU assay to accommodate the higher PPA levels expected in PKU urine (from 500–12,000 μM when plasma concentrations exceed 0.25 mM [19, 34]). We found the best results were obtained when urine samples were diluted 4X with water. When the colored products were scanned from 300 to 700 nm, PPA had the same absorbance maxima as pyruvate and KIC (430 nm) and KIC, KIV, KMV, and AKG (530 nm) (Figure S1). When 4X diluted urine sample was spiked with 100 μM pyruvate and 200 μM AKG (common α‐keto acids in normal urine) and 300–1500 μM phenylpyruvate, creating 300–1800 μM total α‐keto acids and assayed, similar reddish‐amber to dark amber products were seen as was observed with MSUD α‐keto acids (Figure S4a). As the second well was spiked with pyruvate and AKG but not phenylpyruvate, this absorbance was set as the minimum absorbance expected in normal diluted urine. Linear calibration curves were generated when the concentrations of phenylpyruvate with 300 μM pyruvate and AKG were plotted against A_430_ and A_530_, yielding high *R* ^2^ (Figure S4b). Therefore, our modified DNPH assay quantitatively measures 300–1800 μM phenylpyruvate with a combined 300 μM pyruvate and AKG in 4X diluted urine. Note that when > 1800 μM phenylpyruvate was spiked into the diluted urine, the precipitation of the hydrazone derivative was observed.

### 3.2. Performance of the Assay With MSUD or PKU α‐Keto Acids

A calibration curve for MSUD α‐keto acids was created in a 3X diluted L‐PU samples spiked with 0–1600 μM total of KIC, KIV, and KMV (without pyruvate and AKG) and assayed using the optimized DNPH protocol. The concentrations of KIC, KIV, and KMV varied in the spiked samples (Table S2) since these α‐keto acids are normally present in unequal concentrations in MSUD urine [18, 35]. As shown in Figure 2(a), little to no colored product was detected in the diluted “0” sample, suggesting that no or very low concentrations of other ketones or α‐keto acids were present. When 300–1600 μM of MSUD α‐keto acids was present, a gradient of color was detected, which was also observed when KIC only was spiked into the L‐PU. This suggests that the mixed α‐keto acids generate similar colors with DNPH and NaOH as was observed with a single α‐keto acid, KIC (Figure S2b). When the keto acid concentrations were plotted against background subtracted A_430_ and A_530_, linear calibration curves were generated with almost identical *R* ^2^ (0.99; Figure 2(b)). The SDs were minimal, suggesting that the assay has low variability and high sensitivity (see Table S3). The CV ranged from 0.55% to 6.76% at 430 nm and from 1.98% to 6.04% at 530 nm (Table S3), suggesting that the reactions and the dilutions are precise. While our goal was to develop an at‐home assay for α‐keto acids, our linear calibration curve for MSUD α‐keto acids has sufficiently high *R* ^2^ and low CVs and SDs that suggest our assay could be used in the laboratory to robustly quantitate α‐keto acids (Table 1).

FIGURE 2(a) MSUD keto acid colorimetric reactions in local pooled urine (L‐PU) at different concentrations. (b) Keto acid calibration curve at both A_430_ and A_530_ (performed in triplicate). (c) Assay vs theoretical correlation graphs for MSUD keto acid assays at both A_430_ and A_530_.

**Figure:**
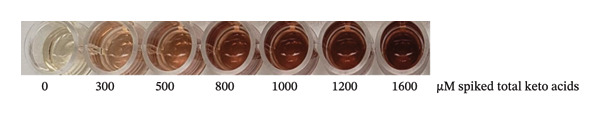
(a)

**Figure:**
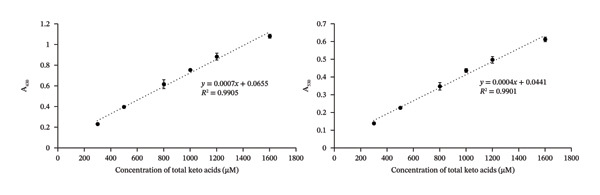
(b)

**Figure:**
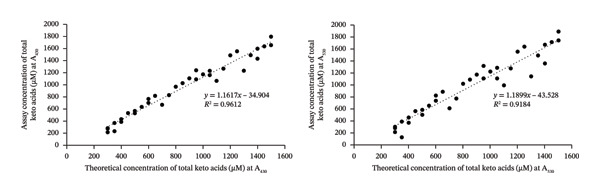
(c)

**TABLE 1:** Linearity, range, LOD, and LOQ of MSUD or PKU assays.

Calibrants	Regression equation	*R* ^2^	Linear range (μM)	LOD (μM)	LOQ (μM)	CV as % (average of all calibrants)
MSUD calibrants in 3X diluted L‐PU A_430_	*Y* = 0.0007*x* + 0.0655	0.9905	300–1600	31	103	3.5
MSUD calibrants in 3X diluted L‐PU A_530_	*y* = 0.0004*x* + 0.0441	0.9901	300–1600	27	89	3.7
PKU calibrants in 4X diluted L‐PU A_430_	*y* = 0.0007*x* + 0.089	0.9865	300–1600	31	103	4.7
PKU calibrants in 4X diluted L‐PU A_530_	*y* = 0.0004*x* + 0.0677	0.982	300–1600	27	89	4.0
MSUD calibrants in C‐PU diluted 1/20 (at‐home assay)	*y* = 0.0001*x* + 0.018	0.9991	575–6200	161	536	1.4
PKU calibrants in C‐PU diluted 1/20 (at‐home assay)	*y* = 0.0002*x* + 0.0169	0.9997	700–7200	81	268	2.1

A calibration curve for phenylpyruvate was generated in the L‐PU sample diluted 4X and spiked with 300–1600 μM phenylpyruvate and assayed using the modified DNPH reaction, performed in triplicate. The reactions and the resultant calibration curves are shown in Figure 3(a) and 3(b), respectively. As SDs were minimal, these suggest that the assay has low variability and high sensitivity (Table S4). The CVs for 300–1600 μM phenylpyruvate measured absorbances at 430 nm and at 530 nm were all < 6%, suggesting that the reactions and dilutions are precise (Table S4, Table 1).

FIGURE 3(a) Phenylpyruvic acid (PPA) colorimetric reactions in local pooled urine (L‐PU) at different concentrations (in triplicate). (b) PPA calibration curve at both A_430_ and A_530_. (c) Assay vs theoretical correlation graphs for the PPA assay at both A_430_ and A_530._

**Figure:**
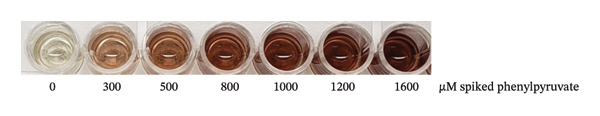
(a)

**Figure:**
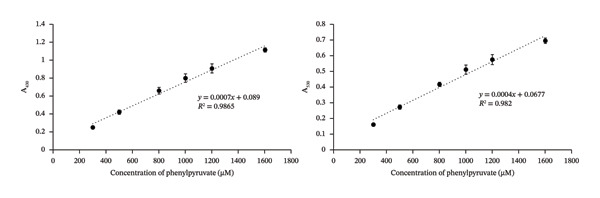
(b)

**Figure:**
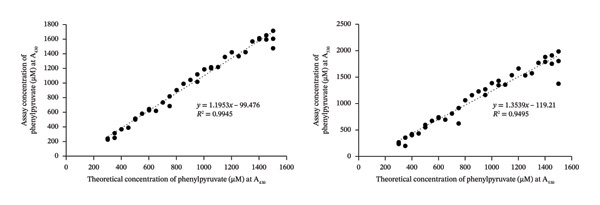
(c)

Our calibration curves were prepared by spiking diluted urine samples with the MSUD or PKU α‐keto acids and subtracting the endogenous background reactions of other minor ketones, and α‐keto acids with the DNPH. However, this subtraction would not be possible with spectrophotometric measurements of patient samples, expected to contain high concentrations of α‐keto acids. We determined the background absorbance values of the DNPH reagent reacted with water and then NaOH. The average absorbance of the DNPH + NaOH reaction alone was 0.144 at 430 nm and 0.071 at 530 nm. These background absorbance values can be subtracted from the absorbance values of patient samples reacted with DNPH/NaOH, and then, net absorbance can be used to determine the concentrations of α‐keto acids in those samples. From blank measurements, the LOD and LOQ of the laboratory assay were calculated to be 31 μM and 103 μM at 430 nm, and 27 μM and 89 μM at 530 nm, respectively, using the slope of the MSUD and PKU calibration curves (which were identical at each wavelength: 0.0007 at 430 nm and 0.0004 at 530 nm).

### 3.3. Validation of the DNPH Assay Using MSUD or PKU Variably Spiked Urine Samples

To mimic the high α‐keto acid levels expected in MSUD or PKU urine samples, several healthy volunteer samples were diluted and then spiked with or without different levels of MSUD α‐keto acids or PPA, respectively, and assayed using the modified DNPH assay. We prepared mock MSUD or PKU urine samples (from healthy volunteer urines) mimicking the α‐keto acid levels expected in MSUD or PKU because the acquisition of authentic MSUD or PKU urine samples from metabolically decompensated or untreated patients is extremely difficult. Instead, we consulted the Human Metabolome Database (HMDB), which provides diagnostic reference ranges for all detected metabolites associated with > 300 IEMs (in blood and urine), including MSUD and PKU [4]. Using these HMDB values, we spiked all known MSUD or PKU organic acids, spanning the ranges expected for untreated MSUD or PKU, to these healthy urine samples [4]. The net absorbance values were then used to determine the assayed concentrations of the spiked α‐keto acids from the calibration curve shown in Figure 2(b) for MSUD and Figure 3(b) for PKU. These assayed concentrations were then plotted against the theoretical spiked concentrations, creating the scatter plot shown in Figure 2(c) for MSUD and Figure 3(c) for PKU. For MSUD, the *R* ^2^ values at 430 nm and 530 nm were 0.96 and 0.92 (Figure 2(c)), respectively, and for PKU, the *R* ^2^ values at 430 nm and 530 nm were 0.99 and 0.95 (Figure 3(c)), respectively, suggesting a strong correlation of the assay‐determined concentrations to the theoretical concentrations. Because urine samples were diluted by 3X for MSUD and the calibration curve detects 300–1800 μM of α‐keto acids, this suggests that three times more MSUD keto acids (up to 4800 μM) can be accurately quantified in urine using this method. Because urine samples were diluted 4X for PKU and the calibration curve detects 300–1600 μM phenylpyruvate, up to 4X more phenylpyruvate (up to 6400 μM) can be accurately quantified in urine samples of PKU patients using this method. For urine samples containing > 4800 μM MSUD keto acids or > 6400 μM phenylpyruvate, further dilutions may be required to ensure measured values fall within our MSUD or PKU calibration curves.

### 3.4. Development of a Portable At‐Home Assay for MSUD and PKU Using Caprolactam

The DNPH‐NaOH assay we developed works fine in a laboratory setting where access to pipettors and UV spectrophotometers is routine. However, to make such an assay work at home, where minimal equipment is available, the assay would need further modifications. A paper‐based or dry assay would be the simplest. However, the DNPH reagent is explosive when dried and therefore unsuitable for at‐home use. Many MSUD patients and families are familiar with, and have used, the liquid DNPH reagent alone at home and would simply need to add NaOH to complete our modified reaction. To minimize errors from adding drops of liquid NaOH, we loaded defined amounts of NaOH into spectrophotometric cuvette caps by freeze‐drying and air‐drying and then determined whether the dried NaOH could be redissolved with the DNPH reagent. We found that NaOH required up to a week to completely air dry, which was far too time consuming. The freeze‐dried NaOH did not completely dissolve (data not shown) likely because the carbonates in the anhydrous NaOH interfered with solubilizing dried NaOH. We looked for other means to immobilize the NaOH and found, after much testing, that ε‐caprolactam, a solid, transparent, and colorless compound, could serve as the immobilizing agent. Gently heated caprolactam melted quickly and immobilized premeasured solid NaOH in cuvette caps as it solidified quickly at RT.

As spectrophotometric assays for α‐keto acids and phenylpyruvate were initially prepared in very small volumes suitable for 96‐well plates for lab settings, we increased the volumes of our reactions to 5.5 mL for easier at‐home handling. We wanted to create readily discernible visual reference color guides for these prototype MSUD and PKU kits. We performed a series of dilutions (3X, 4X, 6X, 10X, and 20X) of a range of concentrations of MSUD keto acids (0–6000 μM) and PKU keto acids (0–6400 μM) expected in urine and found that a 20X dilution worked best, showing a linear response and providing the clearest gradient with the most distinction between colors for both MSUD and PKU (Figure S5).

To prepare the color reference guide, we prepared serial dilutions of MSUD keto acids (375–6000 μM‐containing equal amounts of KIC, KIV, KMV plus 200 μM AKG spiked into each data point, excluding 0 μM, making the total keto acids 575 – 6200 μM) and phenylpyruvate (393–6776 μM in urine plus 210 μM–218 μM AKG—determined by NMR, spiked into each data point, excluding 0 μM, making the total keto acids 603–6994 μM) and reacted these solutions with the at‐home assay (Figures 4(a) and 4(b)). When A_530_ measured for the MSUD standards were plotted against the theoretical levels of the MSUD‐spiked‐in keto acids, a linear calibration curve with a high *R* ^2^ (0.9991; Figure 4(c)) and low CV (1.4%, average of all calibrants; Table 1) was obtained. For the PKU standards, the concentrations of PPA and AKG in each of the PKU standards were quantified by NMR and summed (Figure 4(b)) and plotted against the absorbance of the at‐home assay, also generating a linear curve with a high *R* ^2^ (0.9997; Figure 4(d)) and low CV (2.1%, average of all calibrants; Table 1). Using the slope of the MSUD calibration curve, the LOD and LOQ were calculated to be 161 μM and 536 μM, respectively. For the PKU calibration curve, the LOD and LOQ were 81 μM and 268 μM, respectively. These LODs and LOQs, calculated from blank determinations, are higher than those reported for the laboratory assay, likely due to the use of a higher DNPH concentration (10 mM compared to 4.4 mM; Table 1). The colored images, converted to square color chart hex value images, are shown in Figures 4(e) and 4(f) and approximate the colors seen in each photograph. To simplify patient readings, the color reference guides were rounded to 600 – 6000 μM for MSUD and 600 – 7000 μM for PKU, ensuring ease of interpretation while maintaining accuracy in estimating α‐keto acid concentrations in urine. This demonstrated that concentrations of the α‐keto acids in the urine of PKU or MSUD patients could be easily determined by comparison with the color guide provided with the prototype kit. We also determined that caprolactam did not alter the chemical reaction as the colors produced with and without caprolactam were identical (data not shown).

FIGURE 4In‐house assay for MSUD and PKU utilizing the assay kit components, including NaOH stopper caps, DNPH, and 7‐mL vials. (a) Photographs of the colored reaction products for the MSUD assay (spiked‐in levels ranging from 0 to 6000 μM, shown on the bottom). (b) Photographs of the colored reaction products for the PKU assay (nuclear magnetic resonance or NMR‐determined concentration levels of PPA and AKG, summed, ranging from 0 to 7000 μM, shown on the bottom). (c) MSUD reactive keto acid standard curve in urine (KIC, KMV, KIV + AKG), showing a linear relationship (*R* ^2^ = 0.9991). (d) PKU reactive keto acid standard curve in urine (PPA + AKG summed but also included L‐phenylalanine, glycine, mandelic acid, which are elevated in PKU patients), showing a linear relationship (*R* ^2^ = 0.9997). (e) MSUD patient color chart and (f) PKU patient color chart, both generated by processing the visual readout using Canva (with the rounded concentrations shown below each chart). Abbreviations: AKG: α‐ketoglutarate; KIC: α‐ketoisocaproic acid; KIV: α‐ketoisovaleric acid; KMC: α‐keto‐β‐methylvaleric acid; MSUD: maple syrup urine disease; NaOH: sodium hydroxide; PPA: phenylpyruvic acid; PKU: phenylketonuria.

**Figure:**
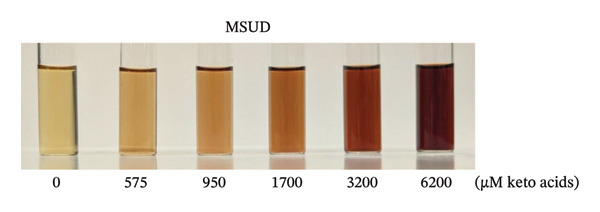
(a)

**Figure:**
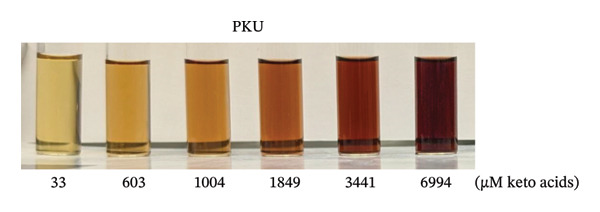
(b)

**Figure:**
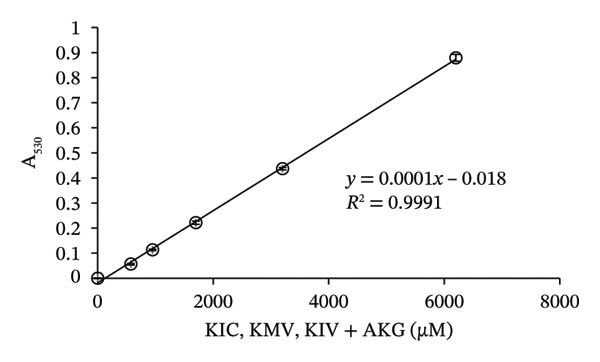
(c)

**Figure:**
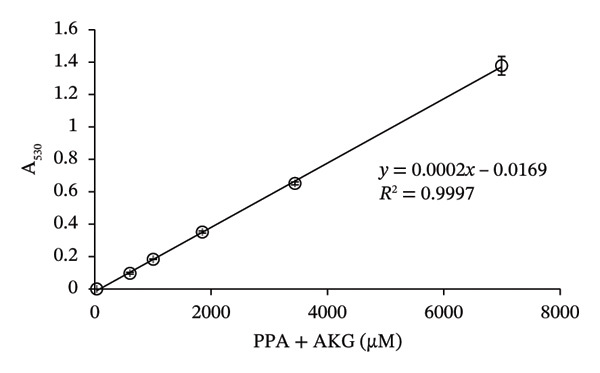
(d)

**Figure:**
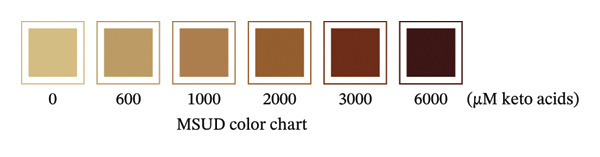
(e)

**Figure:**
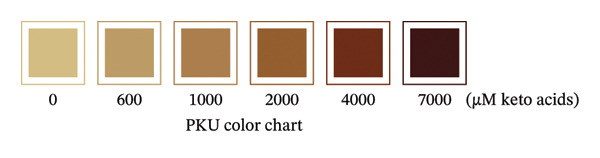
(f)

For simplicity and ease of use, the concentrations shown in the kit’s guide refer to concentrations of α‐keto acids in the neat urine, not the diluted urine samples. The actual concentrations of MSUD α‐keto acids added to DNPH/NaOH to generate the color guide are 30–300 μM, and those for the PKU guide are 30–350 μM. These are less than the concentrations used to generate the calibration curves for the spectrophotometric assays. The concentrations in the spectrophotometric assay were optimized to generate absorbance values from 0.1 to 1.0 to ensure accurate readings using a spectrophotometer. The concentrations and dilutions for the color guide were set lower to produce more readily discernible color ranges for at‐home use and are relevant to ranges expected in MSUD and PKU patients.

After finalizing the protocols, testing, color chart generation, and assay storage requirements, we prepared a prototype at‐home kit for the detection of both the MSUD and PKU α‐keto acids (Figure S6). If the kits move beyond the prototype stage, they will have to be packaged and shipped to locations around the world. Therefore, we determined whether the caprolactam–NaOH was stable over long periods and produced identical results when stored at RT. We mixed the liquid DNPH solution with unspiked (0 μM) and spiked (1500 μM, containing 500 μM each of KIC, KIV, and KMV) urine samples and then, after 5 min, added caprolactam–NaOH cuvette caps stored for 1, 30, and 60 days at RT (covered by parafilm). The color produced after 30‐ and 60‐day storage was identical to that after one day of storage (data not shown), confirming that caprolactam–NaOH is stable at RT for at least two months and should not affect the sensitivity of the at‐home assay.

We also examined the stability of the DNPH reagent to determine whether identical results could be seen with storage at RT compared to 4°C. We mixed unspiked (0 μM) and PPA‐spiked (4000 μM) C‐PU thawed aliquots with DNPH stored in vials at RT or 4°C and, after 5 min, added the caprolactam–NaOH caps. Using S/N of the A_430_ of 4000 μM compared to 0 μM, the S/N was variable for the DNPH stored at RT (Figure S7). At 4 weeks of storage at RT, precipitates were seen that were not observed in the DNPH stored at 4°C. For 4–12 weeks, the S/N of the RT‐stored DNPH continued to decrease (Figure S7). In contrast, for DNPH stored at 4°C, the S/N was unchanged at 1–3 weeks and at 12 weeks. The S/N increased slightly at 4 and 8 weeks. Despite these differences, the interassay variability of the absorbance values of the spiked samples (*n* = 7) over 12 weeks was low (CV = 4.3%: Table S1). These results demonstrated that the DNPH reaction vials must be stored at 4°C to maintain consistent reaction performance and to ensure the results remain comparable over the 12‐week period.

### 3.5. Other Keto Acids Reacting With the DNPH in the At‐Home Assay

Urine is a complex matrix containing several classes of chemicals with keto functional groups that could react with DNPH in the at‐home assay, producing color in addition to KIV, KIC, KMV, AKG, and pyruvate for MSUD and PPA, AKG, and pyruvate for PKU (that have already been discussed). We tested acetone (0–1200 μM in water) and acetaldehyde (0 – 500 μM in water) and saw increased absorbances at A_530_ for both compounds with acetaldehyde yielding higher absorbances with lower concentrations than seen with higher levels of acetone (Figure S8a). However, no acetone and acetaldehyde were detected or quantified in the four PKU samples (Table 2) and thus were not included in the standard PKU solutions prepared for the calibration curves. We also tested 500 μM each of OAA, phenylacetic acid, and 4‐HPAA using the at‐home assay at the same time we assayed AKG, PPA, and pyruvate. When A_530_ for PPA was compared to the absorbances of the other metabolites, absorbance changes were seen, ranging from 42.3% of PPA for AKG to 93.8% of PPA for OAA (Table S5). This suggests that these keto acids could contribute to additional absorbance in urine samples. However, visible color changes varied despite 500 μM being tested for each while no color change was discernible for phenylacetate (Figure S8b), suggesting that each of these metabolites may not contribute an equivalent amount of color as PPA. We also tested phenylalanine, phenyllactate, and 2‐hydroxyphenylacetate, but no change in absorbance or color was seen with these metabolites.

**TABLE 2:** Concentrations of metabolites quantified by nuclear magnetic resonance (NMR) spectroscopy compared to the at‐home keto acids assay measured in adults.

Metabolite	HMDB number	Expected levels (normal, μM)	Expected levels (PKU, μM)	Concentration (μM)				
				C‐PU	PKU before breakfast	PKU after breakfast	PKU after dinner	PKU after lunch
3‐Phenyllactate	HMDB0000779	2.3–4.9 [4]	313 ± 116^a^ [4]	0.0	37.3	37.3	37.3	37.3
3‐Indoleacetic acid	HMDB0000197	15–28 [4]	37.5–528.8^a^ [36]	0.0	35.0	0.0	46.6	23.9
4‐Hydroxyphenyl‐pyruvic acid (4‐HPPA)	HMDB0000707	16.5 (1.5–87.4) [4]	24.47–5374^b^ [4]	0.0	13.6	53.0	34.1	92.4
Acetate	HMDB0000042	130 (25–1060)^c^ [4] 89 ± 72 [29]	163–3753^c^ [4] 172 ± 133 [29]	115	100	71.6	85.3	69.4
Acetone	HMDB0001659	2–861.4 [4]	Normal	56.8	0.0	0.0	0.0	0.0
Acetaldehyde (aldehyde or hydrate)	HMDB0000990^d^	26 (8–42) [4]	Normal	0.0	0.0	0.0	0.0	0.0
Creatinine	HMDB0000562	500–35,000 [4]	3938–6112 [4]	6255	13,022	10,242	10,606	7892
Glycine	HMDB0000123	106 (44–300)^c^ [4] 1178 ± 1241 [29]	822–3048^c^ [4] 2826 ± 3422 [29]	1161	1968	3346	1499	2480
Hippurate	HMDB0000714	229 (19–622) [4]	Normal	1353	1692	1030	1019	966
Mandelate	HMDB0000703	14 (11–17) [4]	99 ± 82 [29]	8.1	117	55.8	21.3	209
Oxaloacetic acid (OAA)	HMDB0000223	22 (12–59) [4]	388 ± 251 [29]	8.1	9.1	26.1	13.5	16.8
*o*‐Hydroxyphenyl acetate	HMDB0000669	20 (9–45) [4]	365.3–973.5^b^ [4]	0.0	200	115	185	48.0
Phenylacetic acid (PAA)	HMDB0000209	41.6 ± 9.1^c^ [4] 55 ± 56 [29]	165–556^b^ ^,^ ^c^ [4] 139 ± 147 [29]	0.0	106	106	106	106
Phenylalanine	HMDB0000159	64 (35–112)^c^ [4] 210 ± 183 [29]	2532–4148^b^ ^,^ ^c^ [4] 397 ± 214 [29]	57.0	434	452	503	543
Phenyl glyoxylate	HMDB0001587	366 ± 4.8 [4]	NA	0.0	17.4	17.4	17.4	24.4
Phenylpyruvic acid (PPA)	HMDB0000205	2.4 (1.0–7.6^c^ [4] 108 ± 102 [29]	1466–310,478^b^ ^,^ ^c^ [4] 739 ± 1266 [29]	0.0	196	319	290	678
Pyruvate	HMDB0000243	27 (8–64) [4]	Normal	5.4	29.1	57.3	30.8	48.0
Alpha‐ketoglutarate (AKG) (2‐oxoglutaric acid)	HMDB0000208	48 (20–170) [4]	Normal	32.5	186	511	251	429
Keto acids—NMR (μM) *PPA + AKG*	NA	NA	NA	32.5	382	830	541	1107
Keto acids—NMR (μM) *PPA + AKG + OAA* + *PAA* + *4-HPPA + pyruvate*	NA	NA	NA	46.0	540	1072	725	1370
Keto acids—at‐home assay (μM) (mean ± SD, A_530_ results)	NA	NA	NA	84.5 ± 26.5	586 ± 25.2	1018 ± 20.8	766 ± 48.1	1328 ± 25.2
Keto acids—NMR (μM/mM creatinine)	NA	NA	NA	7.4 μM/mM	41.5 μM/mM	104.7 μM/mM	68.4 μM/mM	173.6 μM/mM
Keto acids—at‐home assay (μM/mM creatinine)	NA	NA	NA	13.5 μM/mM	45.0 μM/mM	99.4 μM/mM	72.2 μM/mM	168.3 μM/mM

Abbreviation: NA, not available or not applicable.

^a^Reported for uncontrolled children in HMDB.

^b^Reported for uncontrolled children and newborns in HMBD.

^c^When levels in Cannet et al. 2023 [29] or the C‐PU or PKU urine samples do not agree with HMDB values, both ranges were reported.

^d^Distinction between aldehyde and hydrate not stated in HMDB.

### 3.6. Validation of the At‐Home Assay Using Clinical Samples and NMR Spectroscopy

Frozen aliquots of the four PPA samples (taken before and after food consumption), assayed in triplicate, were measured by the at‐home assay (Figure 5(a)) and by NMR (Table 2; see Figure S9 of ^1^H‐NMR spectra). When the assayed determined concentrations of keto acids were plotted against the NMR‐determined concentrations of PPA plus AKG, a high *R* ^2^ (0.997; data not shown) was produced. This suggests that the at‐home assay can accurately detect the keto acids in these clinical PKU samples collected with and without diet restriction. However, NMR levels were 200 μM lower than the assay‐determined concentrations (Table 2). As reported in the above section, oxalacetate, 4‐HPPA, phenylacetate, and pyruvate also produce color when reacted with DNPH. When the concentrations of these metabolites were summed and the NMR‐determined concentrations of PPA, AKG, OAA, 4‐HPPA, phenylacetate, and pyruvate (Table 2) were plotted against assay‐determined concentrations, a high *R* ^2^ = 0.9944 was obtained (Figure 5(b)).

FIGURE 5Comparison of nuclear magnetic resonance (NMR)–determined concentrations of keto acids in four clinical PKU samples quantified by NMR to the at‐home assay levels. (a) Photograph of the colored products seen when four clinical PKU patient urine samples (obtained at different times throughout the day) were reacted via the at‐home assay in reaction vials with their summed PPA and AKG (+ others reactive metabolites) NMR‐determined sample concentrations. (b) Correlation of the at‐home assay concentrations (extrapolated from the standard curve equation of the NMR calibration curve shown in Figure 4(d)) against the NMR levels (PPA + AKG + further reactive metabolites determined within samples including OAA + 4‐HPPA + pyruvate + phenylacetate) quantified in the four PKU urine samples. The PKU patient samples, measured concentrations before breakfast (following an overnight fast), after breakfast, after lunch, and after dinner, showed fluctuations in metabolite levels in response to food intake. Error bars represent the standard deviation (SD) from triplicate measurements. c, d) Bland–Altman plots of levels of keto acids quantified by NMR compared to the at‐home assay expressed in μM (c) and normalized to creatinine concentrations ((d), μM/mM) showing mean differences and upper and lower 1.96 × SD levels of agreement. Abbreviations: AKG: α‐ketoglutarate; 4‐HPPA: 4‐hydroxyphenylpyruvic acid; OAA: oxaloacetic acid; PPA: phenylpyruvic acid; PKU: phenylketonuria.

**Figure:**
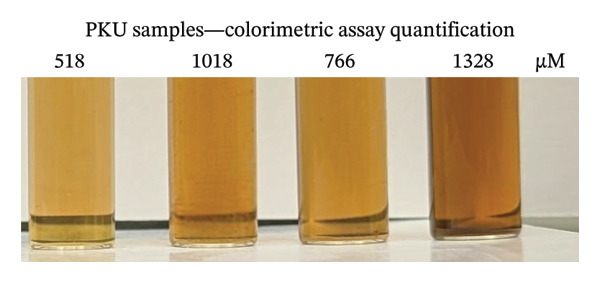
(a)

**Figure:**
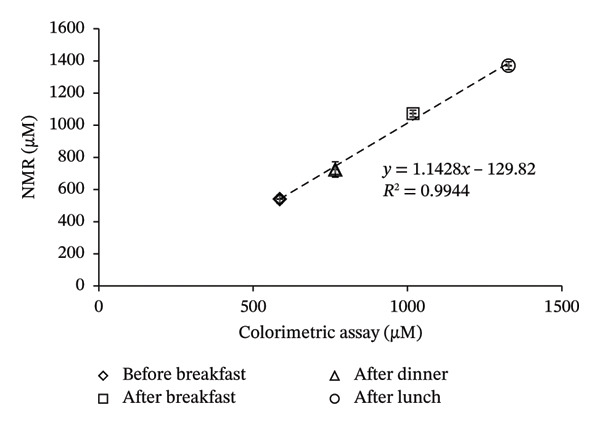
(b)

**Figure:**
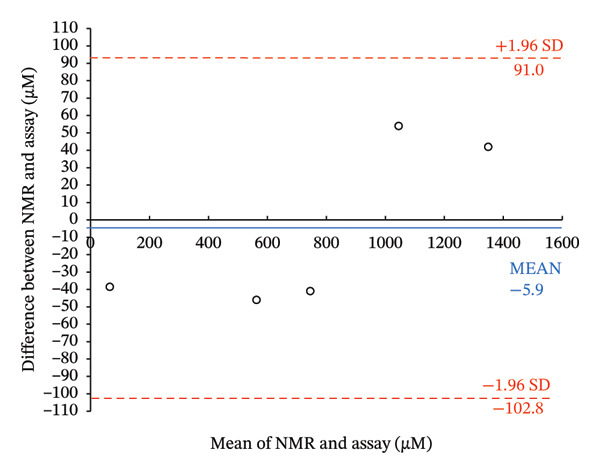
(c)

**Figure:**
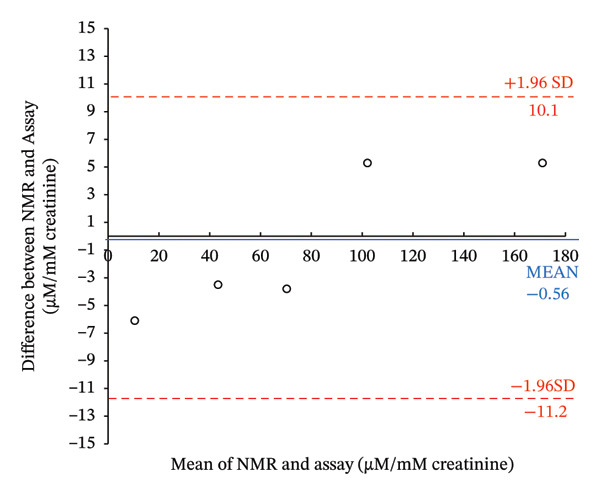
(d)

We noted earlier that L‐phenylalanine, glycine, and mandelic acid were included in our PKU standards as they were reported to be significantly elevated in PKU urine [29]. We also observed that these metabolites were also increased significantly in the PKU clinical samples (Table 2) compared to the C‐PU (which was obtained from 12 healthy donors [27]). Other keto acids that were quantified in the PKU clinical samples but undetectable (below the LOD) in C‐PU included 4‐HPPA, *o*‐hydroxyphenylacetate, 3‐phenyllactate, phenylacetic acid, and phenyl glyoxylate. Pyruvate and AKG were also increased in the PKU samples compared to C‐PU. Even though many keto acids were quantified by NMR, only the 4‐HPPA, OAA, and phenylacetate, in addition to AKG and pyruvate, would be contributing to the color seen with the at‐home assay.

Creatinine was also quantified by NMR in these samples (Table 2). Most assay‐derived creatinine‐normalized levels approximated NMR creatinine‐normalized levels except for those measured for the C‐PU samples. Creatinine levels were within the normal range (7.9–10.2 mM) in three PKU samples and were higher in the sample collected after an overnight fast (13.0 mM). Despite this variation, the NMR‐normalized concentrations remained consistent with the assay‐determined concentrations, suggesting that urinary keto acid measurements using this assay do not require creatinine normalization.

A Bland–Altman analysis was conducted to evaluate the agreement between NMR‐determined and our colorimetric assay‐determined keto acid concentrations with or without creatinine normalization. For the Bland–Altman plot of concentrations not normalized to creatinine (Figure 5(c)), the mean bias was −5.9 μM with all measurements falling well within the 1.96 SD limits of agreement or LOA (−102.8 μM–91.0 μM). For the Bland–Altman plot of concentrations normalized to creatinine (Figure 5(d)), the mean bias was −0.56 μM/mM creatinine with all measurements falling well within the 1.96 SD limits of agreement or LOA (−11.2 μM/mM to 10.1 μM/mM). These creatinine‐normalized values were approximately 10X less than the values seen when concentrations were not normalized to creatinine. The analysis included five urine samples, consisting of four different PKU samples and one normal urine sample (C‐PU). The differences were generally centered around the mean, indicating no significant systematic bias between the methods.

## 4. Discussion

MSUD and PKU are IEMs arising from defects in enzymes for metabolizing BCAA (leucine, isoleucine, and valine) and phenylalanine, respectively. These enzymatic deficiencies lead to an accumulation of these amino acids and their α‐keto acid by‐products in blood, which can travel to the brain, disrupting neurotransmitter balances, resulting in dire neurological consequences. Even patients adhering to restrictive diets remain vulnerable to metabolic decompensation triggered by infection, surgery, and/or stress, which can cause dangerous spikes of these harmful amino acids or α‐keto acids [37]. Continuous monitoring is essential to maintain these compounds within clinically safe ranges [31, 32, 38, 39]. Currently, many MSUD families use the nonquantitative, at‐home DNPH spot test to monitor urinary α‐keto acid levels, but its results are difficult to interpret. For PKU patients, the only at‐home option—keto‐sticks—measures acetoacetate rather than PPA. These limitations motivated our team to develop a more robust, quantitative multimetabolite assay for detecting urinary α‐keto acids.

This study presents a modified, two‐step quantitative colorimetric assay that enables the rapid detection of multiple urinary keto acids in MSUD and PKU patients. By adding NaOH to the original DNPH reaction, the assay eliminates the formation of a yellow precipitate, producing a clear, amber‐colored solution that is easy to interpret. The assay quantitatively measures keto acid concentrations ranging from 700 to 7200 μM within 10 min, offering a significant improvement over the existing qualitative DNPH assay (Brady’s test). Correlation studies demonstrated high coefficients of determination for MSUD‐spiked urine and PPA‐spiked urine. This quantitative colorimetric multimetabolite assay for urinary keto acids addresses the limitations of the existing qualitative assay (Brady’s test), providing clear and interpretable results for both MSUD and PKU. This makes it a valuable contribution to metabolic disorder diagnostics and monitoring. Collectively, four key modifications allowed us to convert a difficult‐to‐interpret at‐home assay into a far more user‐friendly, more easily interpreted, quantitative assay that is more portable and more reliable: (1) adding NaOH to the reaction to enhance color contrast; (2) using urinary dilution to minimize interference; (3) applying melted caprolactam for precise delivery of NaOH; and (4) creating a color chart to avoid the need for a spectrophotometer.

We also validated the at‐home assay using clinical PKU samples by comparing its results to NMR‐determined keto acid concentrations. The assay reliably detected keto acids in these samples, with concentrations closely matching NMR‐derived values even when samples were collected before and after food consumption. Furthermore, our analysis demonstrated that creatinine normalization did not significantly impact the assay’s accuracy, suggesting that it may not be necessary for routine use. The assay specifically detected keto acids (PPA, AKG, 4‐HPPA, OAA, pyruvate, and phenylacetic acid) in PKU samples, reinforcing its specificity. Bland–Altman analysis showed a high level of agreement between the at‐home assay and NMR measurements, supporting its potential as a reliable at‐home monitoring tool. Unfortunately, we were unable to acquire and test authentic MSUD urine samples due to the rarity of MSUD in our local patient population. Furthermore, authentic MSUD urine samples were not commercially available, and routine monitoring in our local clinic typically relies on plasma rather than urine, preventing us from acquiring and analyzing authentic MSUD urine samples as thoroughly as authentic PKU urine samples.

Because home users do not have at‐home spectrophotometers, we modified the assay so that the colors generated by the assay could be readily distinguished and easily compared via a paper color chart. We accomplished this by diluting urine samples 20X for the at‐home colorimetric multimetabolite assay. To make the prototype at‐home kit easy to ship and easy to store, we discovered that consistent, dry NaOH formulation could be achieved by using caprolactam, which allowed us to create a prototype kit with reagents consisting of vials preloaded with acidic DNPH (which is stable for long periods of time at 4°C) and preweighed NaOH caps. Caprolactam has been widely used in the production of nylon fibers and in engineering resins and films [40]. However, to our knowledge, this is the first reported use of caprolactam as a formulation reagent and this success suggests that caprolactam could be applied to other assays requiring a consistent delivery of inorganic bases. Our colorimetric multimetabolite assay could potentially be modified to detect other keto acids that build up in the urine of other genetic disorders. These include IEMs such as tyrosinosis Type 2 and tyrosinemia where increased plasma tyrosine results in the accumulation of 4‐hydroxyphenylpyruvic acid; histidinemia, which is characterized by increased imidazolepyruvic acid; and methionine malabsorption or oast house syndrome, which is characterized by increased levels of α‐ketobutyric acid [9, 41].

Additionally, we are in the process of obtaining ethical approval to evaluate the assay in real‐world clinical settings, using urine samples collected from PKU and MSUD patients during routine DBS testing. The goal is to determine whether the at‐home assay could serve as a proxy for delayed DBS results, which typically take five to 6 days to process. Clinicians are particularly interested in adopting this novel colorimetric assay due to its rapid turnaround time, potentially allowing patients to monitor their metabolic status within minutes rather than days. We are also working on developing a smart‐phone application equipped with an RGB sensor to automatically read and interpret assay results, thereby reducing interuser variability in color‐based interpretation during home use.

We also demonstrated that our colorimetric multimetabolite assay is suitable for the laboratory spectrophotometric quantification of up to 4800 μM for branched‐chain α‐keto acids and up to 6400 μM for PPA, with both low LODs and LOQs (MSUD LOD 31 μM, LOQ 103 μM; PKU LOD 27 μM, LOQ 89 μM). By using modest dilutions, linear calibration curves for branched‐chain α‐keto acids and PPA were achieved yielding much more reproducible results across a wide range of urine backgrounds spiked with a broad range of different α‐keto acids. While the assay was designed to detect the elevated concentrations, the LODs and LOQs of both the laboratory and at‐home formats (MSUD: LOD 161 μM, LOQ 536 μM; PKU: LOD 81 μM, LOQ 268 μM) fell below the dynamic range of 700–7200 μM, which encompasses clinically relevant concentrations observed during metabolic decompensation.

It is important to note that there are other colorimetric methods that can detect α‐keto acids in urine samples. These methods use ferric chloride [42], diazonium chemistry [43], nitroprusside [43–46], or chemical derivatization [47, 48]. For instance, PPA can be detected by diazonium chemistry [43] using p‐chloroaniline [49]. However, this reaction uses many harsh reagents, has multiple, time‐consuming steps, and does not produce color with the α‐keto acids known to be in MSUD. The Salkowski reagent, FeCl_3_ in sulfuric acid, can detect indole‐3‐pyruvate [50], but it requires a solvent extraction step, which is not compatible for at‐home testing. In another assay, hydroxylamine hydrochloride, combined with FeCl_3_, can convert various α‐keto acids to hydroxamic acids that will form a colored complex with FeCl_3_ [51]. However, hydroxylamine hydrochloride is toxic and unsuitable for at‐home testing. Sodium nitroprusside [42, 44–46], which is found in commercial urine test strips (keto‐sticks), detects acetone, acetoacetate (a β‐keto acid) [42], and methyl ketones [44]. However, only KIC of the MSUD α‐keto acids will be detected with sodium nitroprusside as it has a methylene group (CH_2_) next to the ketone carbonyl and will yield a positive test result. Moreover, the sodium nitroprusside reaction lacks specificity for methylketones as it also reacts with thiols [45] and amines (alkaloids and drugs) [46], which may be present in urine. In addition to these chemical assays, several high‐performance liquid chromatography (HPLC) methods have been shown to detect α‐keto acids and/or phenylpyruvate in human urine, serum, or plasma samples. These use chemiluminescence after derivatization with 4, 5‐diaminophthalhydrazide [48] or employ fluorescence after derivatization with 4′‐hydrazino‐2‐stilbazole [47]. These assays could be applied to quantify phenylpyruvate in urine in a well‐equipped laboratory but could not be used in an at‐home test since they require costly chemiluminescent or fluorescent detectors. None of the above‐mentioned methods specifically detect the targeted α‐keto acids, KIC, KIV, and KMV, that accumulate with MSUD, and only p‐chloroaniline detects PPA. Overall, we believe this NaOH‐modified DNPH test uniquely offers the sensitivity, specificity and ease‐of‐use that would make it an ideal at‐home multimetabolite test for the α‐keto acids that are key to diagnosing and monitoring MSUD and PKU.

## 5. Conclusion

This study introduced a simple, inexpensive colorimetric multimetabolite assay detecting multiple α‐keto acids in urine, demonstrating significant improvements over Brady’s test for MSUD and offering a promising at‐home monitoring approach for PKU. The assay showed strong agreement with quantitative NMR‐based measurements and successfully detected diet‐dependent changes in authentic PKU urine samples, underscoring both its novelty and its potential utility for routine metabolic monitoring of PKU and MSUD patients.

Although the assay performed well under controlled conditions, other factors need to be considered as this test moves toward clinical application. In patients with poorly controlled MSUD, poor diets, fasting, infections, or fever, ketosis leads to elevated urinary acetoacetate and acetone, both of which are DNPH‐reactive and could contribute to increased assay signals. Ketosis also leads to the release of BCAAs from the liver and muscle, further elevating BCAA‐derived keto acids. Consequently, in MSUD patients, this at‐home assay can effectively monitor both ketotic states (brought on by fasting or fever) and metabolic crises (brought on by accidental BCAA consumption). However, this dual application also highlights the need for careful interpretation outside stable metabolic conditions. Limited availability of MSUD clinical samples restricted our ability to evaluate the assay across the full spectrum of MSUD phenotypes, and future work should examine how diet, supplements, ketosis, hydration, or medication, including immunosuppressive drugs such as tacrolimus and prednisone [52] taken by MSUD liver transplant recipients, affect assay accuracy.

While the assay quantifies keto acid concentrations over a wide range, its performance at extremely low or extremely high concentrations requires further validation, particularly for patients experiencing severe metabolic crises or those with well‐controlled disease. Real‐world testing of PKU and MSUD clinical urine samples will be essential to confirm the assay’s robustness across different urine compositions. This assay is not yet formally approved for clinical use and should not be considered a standalone diagnostic tool until further clinical validation is complete.

Given its simplicity, affordability, and noninvasive format, the assay may ultimately support preliminary screening for IEMs such as PKU and MSUD, particularly in regions lacking access to LC‐MS/MS‐based diagnostics. While not intended to replace MS‐based confirmatory testing, continued refinement and comprehensive clinical validation could position this assay as an accessible front‐line tool to flag at‐risk individuals for follow‐up analysis, helping to address critical gaps in global newborn screening infrastructure.

**Nomenclature**
2,4‐DNPH: 2,4‐Dintrophenylhydrazine
4‐HPPA: 4‐hydroxyphenylpyruvic acid
AKG: Alpha‐Ketoglutarate
BCAAs: Branched‐Chain Amino Acids
BCKD: Branched‐Chain α‐Keto Acid Dehydrogenase Complex
BZK: Benzalkonium Chloride
C‐PU: Commercial Pooled Urine
CV: Coefficient of Variation
DBS: Dried Blood Spot
FeCl_3_
: Ferric Chloride
GC‐MS: Gas Chromatography‐Mass Spectrometry
HMDB: Human Metabolome Database
HPLC: High‐Performance Liquid Chromatography
HREB: Human Research Ethics Board
IEM: Inborn Error of Metabolism
KIC: α‐Ketoisocaproic Acid
KIV: α‐Ketoisovaleric Acid
KMV: α‐Keto‐β‐Methylvaleric Acid
LC‐MS/MS: Liquid Chromatography‐Tandem Mass Spectrometry
LOD: Limit of Detection
LOQ: Limit of Quantification
L‐PU: Local Pooled Urine
MSUD: Maple Syrup Urine Disease
NaOH: Sodium Hydroxide
NMR: Nuclear Magnetic Resonance
OAA: Oxaloacetic Acid
PAH: Phenylalanine Hydroxylase
PETG: Polyethylene Terephthalate Glycol–Modified
PKU: Phenylketonuria
PPA: Phenylpyruvic Acid
PU: Pooled Urine
*R* ^2^
: Coefficient of Determination
RT: Room Temperature
SD: Standard Deviation
S/N: Signal to Noise
UV–Vis: Ultraviolet–Visible (Spectroscopy)

## Author Contributions

Conceptualization: Dipanjan Bhattacharyya, Yeganeh Khaniani, and David S. Wishart; formal analysis: Dipanjan Bhattacharyya and Abby Kropielnicki; investigation: Dipanjan Bhattacharyya, Abby Kropielnicki, and Brian L. Lee; methodology: Dipanjan Bhattacharyya, Abby Kropielnicki, Brian L. Lee, and Yeganeh Khaniani; validation: Dipanjan Bhattacharyya, Abby Kropielnicki, Brian L. Lee, and Yeganeh Khaniani; visualization: Dipanjan Bhattacharyya, Abby Kropielnicki, Yeganeh Khaniani, and Marcia A. LeVatte; writing–original draft: Dipanjan Bhattacharyya, Abby Kropielnicki, Yeganeh Khaniani, and Marcia A. LeVatte; supervision: Marcia A. LeVatte and David S. Wishart; writing–reviewing and editing: Marcia A. LeVatte, Abby Kropielnicki, and David S. Wishart; funding acquisition: David S. Wishart; project administration: David S. Wishart; resources: David S. Wishart.

## Funding

This work was supported by the Canada Foundation for Innovation (CFI MSIF #42495), Genome Alberta, a division of Genome Canada, and the Canada Research Chairs Program (CRC Tier 1 #100628).

## Ethics Statement

The study was conducted in accordance with the Declaration of Helsinki and approved by the University of Alberta’s Human Research Ethics Board (HREB) biomedical ethics committee (Protocol Code HREB #Pro00092437, June 3, 2024) for the collection of human urine.

## Consent

Informed consent was obtained from all participants involved in the study.

## Conflicts of Interest

The authors declare no conflicts of interest.

## Supporting Information

Scheme S1: Metabolism of BCAAs, SUPPORTING METHODS: Materials and other consumables, pyruvate‐spiked urine samples, branched‐chain keto acid–spiked urine samples with and without pyruvate and AKG, PPA‐spiked urine samples with and without pyruvate and AKG, preparation of PPA and branched‐chain α‐keto acid calibration curves, preparation of PPA and keto acid correlation curves from spiked samples, preparation of α‐keto acids and PPA standard solutions for standard color charts, quantification of keto acids, and other metabolites by NMR metabolomics. SUPPORTING RESULTS: Initial optimization of the assay protocol using pyruvic acid, assay optimization for MSUD α‐keto acids, performance of the assay using MSUD α‐keto acid–spiked urine containing pyruvate and AKG, Table S1: Stability of study reagents and urine samples and number of replicates used in assays and interassay variability, Table S2: Composition of the keto acids mixtures tested using the DNPH‐NaOH assay and expected ranges in normal and MSUD or PKU patients, Table S3: SD and CV (%) for MSUD keto acid assays, Table S4: SD and CV (%) for PKU phenylpyruvate assay, Table S5: Metabolites that react with DNPH to produce color and their percentage of absorbance relative to PPA, Figure S1: Absorbance maxima of keto acids reacted with DNPH and NaOH, Figure S2: Colored products after reaction of diluted local pooled urine (L‐PU) with DNPH (a) followed by the addition of a base (b), Figure S3: (a) MSUD keto acid colorimetric reaction in the presence of pyruvate and AKG and (b) assay calibration curves, Figure S4: (a) PPA colorimetric reaction in the presence of pyruvate and AKG, Figure S5: Optimal dilution of urine samples needed to generate visually discernible colors and a linear trend using the at‐home assay, Figure S6: PKU/MSUD Test Kit Prototype, Figure S7: Stability testing of the premeasured DNPH stored in the kit vial and dried NaOH stoppers/caps, Figure S8: Metabolites that react with DNPH and NaOH and contribute to the color of the at‐home assay, Figure S9: ^1^H‐NMR spectra from the commercial pooled urine (CPU) and the four PKU urine samples, showing major peaks for AKG, PPA, phenylacetic acid (PAA), and hippuric acid (HA).

## Supporting information


**Supporting Information** Additional supporting information can be found online in the Supporting Information section.

## Data Availability

The data collected and analyzed in the present study are presented in the figures and tables of this manuscript or are provided in the figures and tables in the Supporting Information.
