# Supplementary material for: A Colorimetric Multimetabolite Assay for Quantitative Measurement of Keto Acids in Urine for At‐Home Monitoring of Metabolic Disorders
Source: J Anal Methods Chem. 2026 Feb 27;2026:4116313. doi: 10.1155/jamc/4116313 (PMC12949077; doi:10.1155/jamc/4116313)
Supplement: Supplementary file 1 — Supporting Information Additional supporting information can be found online in the Supporting Information section. [file JAMC-2026-4116313-s001.docx]

Supplementary Materials for

**A Colorimetric Multi-Metabolomic Assay for Quantitative Measurement of Keto Acids in Urine for At-Home Monitoring of Metabolic Disorders**

Dipanjan Bhattacharyya^1⁑^, Abby Kropielnicki^1⁑^, Brian L. Lee^1^, Yeganeh Khaniani^1^, Marcia A. LeVatte^1^, David S. Wishart^1,2,3,4^*

^1^Department of Biological Sciences, University of Alberta, Edmonton, AB, Canada T6G 2E9

^2^Department of Computing Science, University of Alberta, Edmonton, AB, Canada T6G 2E8

^3^Department of Laboratory Medicine and Pathology, University of Alberta, Edmonton, AB, Canada T6G 2B7

^4^Faculty of Pharmacy and Pharmaceutical Sciences, University of Alberta, Edmonton, AB, Canada T6G 2H7

^⁑^ These two authors contributed equally to this work

*Corresponding author: Dr. David S. Wishart Dept. of Biological Sciences, CW-405, Biological Sciences Building, University of Alberta, Edmonton, AB, Canada, T6G 2E8

Telephone: 1-780-492-8574

Email: dwishart@ualberta.ca

**TABLE OF CONTENTS**

p. 2-3 Table of contents

p. 4 Scheme S1. Metabolism of branched chain amino acids.

**pp. 5-11 SUPPLEMENTARY MATERIALS and METHODS**

p. 5 Materials and other consumables

p. 6 Pyruvate spiked urine samples

pp. 6-7 Branched chain keto acids spiked urine samples with and without pyruvate and α -ketoglutarate

pp. 7-8 Phenylpyruvate (PPA) spiked urine samples with and without pyruvate and α-ketoglutarate

p. 8 Preparation of PPA and branched-chain α-keto acid calibration curves

p. 8-9 Preparation of PPA and keto acid correlation curves from spiked samples

p. 9-10 Preparation of α-keto acids and PPA standard solutions for standard color charts

p. 10-11 Quantification of keto acids and other metabolites by nuclear magnetic resonance (NMR) metabolomics

**pp. 12-29 SUPPLEMENTARY RESULTS, TABLES and FIGURES**

pp. 12-13 Initial optimization of the assay protocol using pyruvic acid

pp. 13-14 Assay optimization for MSUD α-keto acids

pp. 14-15 Performance of the assay using MSUD α-keto acids spiked urine containing pyruvate and α-ketoglutarate

p. 16 **Table S1** Stability of study reagents and urine samples and number of replicates used in assays and inter-assay variability

p. 17 **Table S2**. Composition of the keto acids mixtures tested using the DNPH-NaOH assay and expected ranges in normal and MSUD or PKU patients.

p. 18 **Table S3**. Standard deviation and coefficient of variation (CV %) for MSUD keto acids assay.

p. 19 **Table S4**. Standard deviation and coefficient of variation (CV %) for PKU phenylpyruvate assay.

p. 20 **Table S5.** Metabolites that react with DNPH to produce color and their percentage of absorbance relative to phenylpyruvic acid (PPA).

p. 21 **Figure S1.** Absorbance maxima of keto acids reacted with DNPH and NaOH.

p. 22 **Figure S2.** Colored products after reaction of diluted local pooled urine (L-PU) with DNPH (a) followed by addition of a base (b).

p. 23 **Figure S3.** (a) MSUD keto acid colorimetric reaction in presence of pyruvate and α-ketoglutarate and (b) assay calibration curves.

p. 24 **Figure S4.** (a) Phenylpyruvic acid (PPA) colorimetric reaction in presence of pyruvate and α-ketoglutarate and (b) assay calibration curves.

p. 25 **Figure S5.** Optimal dilution of urine samples needed to generate visually discernible colors and a linear response using the at-home assay.

p. 26 **Figure S6.** PKU/MSUD Test Kit Prototype.

p. 27 **Figure S7.** Stability testing of the pre-measured dinitrophenylhydrazine (DNPH) stored in the kit vial and dried NaOH stoppers/caps

p. 28 **Figure S8.** Metabolites that react with DNPH and NaOH and contribute to the color of the at-home assay.

p. 29 **Figure S9.** ^1^H-Nuclear magnetic resonance (NMR) spectra from the commercial pooled urine (C-PU) and the four PKU urine samples, showing major peaks for α-ketoglutarate (AKG), phenylpyruvic acid (PPA), phenylacetic acid (PAA) and hippuric acid (HA).

pp. 30-31 **REFERENCES**
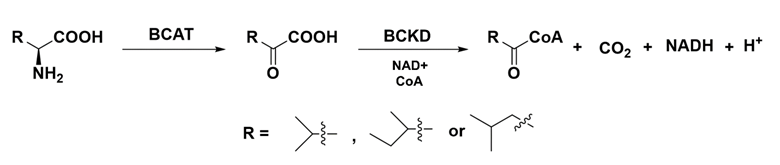


**Scheme S1.** **Metabolism of branched chain amino acids**. L-leucine, L-valine and L-isoleucine are trans-aminated by the branched chain aminotransferase (BCAT) into α-ketoisocaproic acid (KIC), α-ketoisovaleric acid (KIV) and α-keto-β-methylvaleric acid (KMV), respectively. Then KIV, KIC and KMV undergo oxidative decarboxylation, catalyzed by the branched-chain alpha-keto acid dehydrogenase complex (BCKD), NAD+ and CoA, generating acyl-CoAs, CO_2_ and NADH. Any defects in any of the four proteins that make up the BCKD complex (as with MSUD) reduces or eliminates the enzyme’s activity, preventing the breakdown of these BCAAs, causing a build-up of KIC, KIV, and KMV.

**MATERIALS and METHODS (SUPPLEMENTARY)**

***Materials and other consumables***

Pyruvic acid (98%), 2, 4-dinitrophenylhydrazine (97%, 2, 4-DNPH), 2-ketoglutaric acid (98%, also known as α-ketoglutaric acid or AKG), α-ketoisocaproic acid (KIC, 98%), α-ketoisovaleric acid sodium salt (KIV, 95%), DL- α-keto-β-methylvaleric acid sodium salt (KMV), 3-phenyllactate, 3-indoleacetic acid, acetate, acetone, acetylaldehyde, creatinine, glycine, hippurate, oxalacetic acid (OAA), phenylacetic acid, phenylalanine, phenylgloxylate, phenylpyruvic acid sodium salt (98%), *o*-hydroxyphenylacetate, 4-hydroxyphenylpyruvic acid (4-HPAA), potassium phosphate monobasic, potassium phosphate dibasic, DSS-d_6_ (2,2-dimethyl-2-silapentane-5 sulfonate), D_2_O (99.9%), NaOH (pellets, ACS grade, 97%) and 2-oxohexamethyleneimine (99%, known as ε-caprolactam) were purchased from Sigma-Aldrich (Oakville, Canada). Mandelate was purchased from AK Scientific (Union City, USA). 2-chloropyrimidine-5-carboxylic acid (98%) was purchased from ArkPharm (Libertyville, USA). Hydrochloric acid (reagent grade, 37%) was purchased from Caledon Laboratories Ltd (Edmonton, Canada). Prototype kit consumables such as 40 mL plastic disposable urine cups, 12 x 9 x 3-inch black shipping boxes, 16 x 12 x 2-inch foam, 2 oz cobalt blue dropper bottles, glass measuring pipette droppers, 7 mL clear glass vials with screw caps and plastic stoppers were purchased from Amazon, Canada. 0.6 mL PCR strip tubes, microfuge tubes (1.5 mL), falcon tubes (15 mL and 50 mL), BioLite 96 Well Multidish plates and pipette tips were purchased from Thermo-Fischer Scientific (Rochester, USA). The nuclear magnetic resonance (NMR) tubes (3 mm) were acquired from Bruker Ltd. (Milton, Canada). Unless otherwise indicated, solutions were prepared in Milli-Q water.

***Pyruvate spiked urine samples***

To prepare the pyruvic acid-spiked urine samples, 100 μL of urine samples were aliquoted into five 0.6 mL PCR tubes. Then 0, 1, 2, 3 and 4 μL of the urine sample, respectively, were removed from the first five tubes and replaced with the same volumes of 10 mM pyruvic acid, yielding urine samples with 0, 100, 200, 300 and 400 μM pyruvic acid, respectively. Then 50 μL of each of these spiked urine solutions were transferred to clean 0.6 mL PCR tubes. These pyruvic acid spiked urine samples were then assayed for the presence of pyruvic acid using our modified DNPH pyruvic acid assay.

***Branched chain keto acids spiked urine samples with and without pyruvate and α-ketoglutarate***

To prepare the α–keto acid containing urine samples, the local pooled urine (L-PU - a composite of the well-hydrated to dehydrated volunteer urines [1]), samples were first diluted 3X (500 μL of L-PU, 1000 μL of Milli-Q water) and then 50 μL of the 3X diluted L-PU was aliquoted into seven separate PCR tubes. The first tube (tube 1) was designated as the “zero control” or the unspiked sample (although it contains endogenous amounts of normal keto acids). The remaining six tubes (#2-#7) were spiked with 300, 500, 800, 1000, 1200 and 1600 µM of branched-chain α–keto acids (KIC, KIV, KMV). These concentrations were made by removing from tubes #2-#7, 1.5, 2.5, 4, 5.0, 6.0 and 8 μL, respectively, of the 3X diluted L-PU and then replaced with equivalent amounts of 10 mM KIC, 10 mM KIV, and 10 mM KMV (see **Table S1** for specific concentrations of α–keto acids in these solutions). These unspiked (0 μM) and spiked (300-1600 μM) urine samples were then assayed for the presence of α–keto acids using our modified keto acid DNPH assay.

As pyruvate and α-ketoglutarate (AKG) are commonly present α–keto acids in normal urine and both acids react with the DNPH, we prepared spiked 3X diluted L-PU samples with these two common α–keto acids and KIC, KIV and KMV. In particular, 50 μL of 3X diluted L-PU was aliquoted into seven separate PCR tubes. The first tube was the “zero control”. The remaining six tubes (#2-#7) were spiked with 100 μM pyruvate and 200 μM AKG and the final five tubes (#3-#7) were spiked with 300, 500, 800, 1000, 1200 and 1500 μM of the branched-chain α–keto acids (KIC, KIV and KMV). With 300 μM (100 μM pyruvate and 200 μM AKG) added to tubes #2-#7, the final total α–keto acid concentrations in tubes #2-#7 were 300, 600, 800, 1100, 1300 and 1800 μM, respectively (see **Table S1** for the composition of α–keto acids in these solutions). These unspiked (0 μM) and spiked (300 – 1800 μM) urine samples were then assayed for the presence of α–keto acids using our modified keto acid/PPA DNPH assay.

***Phenylpyruvate (PPA) spiked urine samples with and without pyruvate and α****-****ketoglutarate***

To prepare the phenylpyruvate (PPA) spiked urine samples, the L-PU sample was first diluted 4X (500 μL of a urine sample, 1500 μL of Milli-Q water). Then 50 μL of the 4X diluted L-PU was aliquoted into seven PCR tubes with tube #1 designated the ‘zero’ control. The remaining six tubes (#2-#7) were each spiked with 300, 500, 800, 1000, 1200 and 1500 μM PPA by removing, 1.5, 2.5, 4, 5.0, 6.0 and 8 μL of diluted L-PU from tubes #2-#7, respectively and then adding the same volumes of 10 mM PPA to these tubes. These unspiked (0 μM) and PPA-spiked (300-1600 μM) urine samples were then assayed for PPA using our modified keto acid/PPA DNPH assay.

Another group of PPA spiked urine samples were also prepared with 100 μM pyruvate and 200 μM AKG in the 4X diluted L-PU as described above. With 300 μM total pyruvate and AKG added to all tubes except the “0 µM” control, the final α-keto acid concentrations were 300, 600, 800, 1100, 1300 and 1800 μM (see **Table S1** for the composition of the mixtures). These unspiked (0 μM) and PPA-spiked (300 – 1800 μM) urine samples were then assayed for the presence of PPA using our modified keto acid/PPA DNPH.

***Preparation of PPA and branched-chain α-keto acid calibration curves***

The PPA calibration curve was generated using the 4X diluted L-PU sample spiked with 0 to 1600 μM PPA and assayed using the modified keto acid/PPA DNPH assay, performed in triplicate. The MSUD α-keto acid calibration curve was generated similarly where 3X diluted L-PU sample was spiked with 0 to 1600 μM of the branched chain α-keto acids and assayed in triplicate. The absorbance readings were measured at A_430_ and A_530_ for the MSUD α-keto acids and for PPA. The background absorbance (from endogenous keto acids) of the unspiked diluted L-PU sample at each wavelength was subtracted from all the readings and the background corrected absorbance measurements were averaged. The standard deviations (SD), coefficients of variance (CV), and coefficient of determination (R^2^) were calculated using Microsoft Excel. The LOD and LOQ were derived from the measured A_430_ and A_530_ values from a baseline blank (50 μL of Milli-Q water using the same amounts of the reagents from the same stock solutions). The LOD was defined as 3×SD/slope and the LOQ as 10×SD/slope [2]. A linear calibration curve was generated by plotting the theoretical spiked concentrations of PPA or α-keto acids against the averaged background-corrected absorbance measurements.

***Preparation of PPA and keto acid correlation curves from spiked samples***

The 4X diluted, variably spiked PPA urine samples or the 3X diluted, variably spiked branched-chain α-keto acid urine samples were quantified using our modified keto acid/PPA DNPH assay. The unspiked (3X or 4X diluted) urine samples were used as negative controls for the branched-chain α-keto acids or the PPA assays, respectively. The A_430_ and A_530_ (for PPA or for total α-keto acids) of these negative controls were subtracted from the absorbance values of the α-keto acid spiked samples at the corresponding wavelengths. The concentrations of the branched chain α-keto acids or PPA-spiked samples were extrapolated from the linear calibration curves for the total α-keto acids or PPA, respectively. These assay-determined concentrations were then plotted against the theoretical concentrations spiked into the diluted urine samples to generate a correlation curve for PPA or total α-keto acids, respectively.

***Preparation of α-keto acids and PPA standard solutions for standard color charts***

To prepare the MSUD 6000 µM stock solution containing 2000 µM each of KIC, KIV, and KMV, 100 mM stock solutions of each keto acid were first prepared. Then, 60 µL of each 100 mM stock was added to 2820 µL of C-PU, yielding 3000 µL solution containing 6000 µM total MSUD keto acids. To generate an 8000 µM PKU stock solution mimicking the composition of patient samples, the keto acids PPA, L-phenylalanine, glycine, and mandelic acid were included as they were significantly elevated in PKU urine [3]. To create a PKU stock solution containing 8000 µM PPA, 400 µM L-phenylalanine, 6500 µM glycine, and 810 µM mandelic acid, 640 µL of 62.5 mM PPA, 25 µL of 80 mM L-phenylalanine, 250 µL of 130 mM glycine, and 25 µL of 162 mM mandelic acid, respectively, were added to a final volume of 5 mL of C-PU. Then 0, 500, 1000, 2000 and 4000 µM PKU standards or 0, 375, 750, 1500 and 3000 µM MSUD calibrants (prepared in 1 mL, final volume) were made by adding only C-PU (for 0 µM control), or by mixing 62.5 µL, 125 µL, 250 µL or 500 µL, respectively, of the 6000 µM MSUD stock or the 8000 µM PKU stock, with 937.5 µL, 875 µL, 750 µL or 500 µL, respectively, of C-PU. To ensure a physiologically relevant AKG concentration, 200 µM AKG was spiked into all MSUD or PKU calibrants using a 50 mM stock solution (except for the 0 µM control) yielding final keto acid concentrations of 0, 575, 950, 1700, 3200, and 6200 µM for the MSUD standards and 0, 700, 1200, 2200, 4200 and 7200 µM for the PKU standards.

***Quantification of keto acids and other metabolites by nuclear magnetic resonance (NMR) metabolomics***

Samples were prepared for NMR spectroscopy using previously published methods [1]. Aliquots (200 μL) of C-PU, the PPA standards, the 4-HPPA or OAA spiked samples and the four PKU urine samples were mixed with 5 × NMR buffer (50 μL; containing 750 mM potassium phosphate, pH 7.0, 5.00 mM deuterated 2,2-dimethyl-2-silapentane-5 sulfonate (DSS-d_6_), 5.84 mM 2-chloropyrimidine-5-carboxylic acid and D_2_O 54 % v/v in H_2_O). Other than dilution with the highly concentrated phosphate buffer, which helps to neutralize the acidity of urine, the samples were not extracted or manipulated in any way. Metabolite recovery is expected to be 100% with no metabolite loss due to matrix effects. The mixtures were vortexed and centrifuged at 21,000 × *g* for 5 min at 4 °C before analysis. If a precipitate was observed, the liquid portion would be removed without disturbing the pellet. All samples were then loaded into 3 mm NMR tubes.

NMR spectra were recorded on a 700 MHz Avance III HD Bruker (Bruker Biospin, Rheinstetten, Germany) NMR spectrometer equipped with a cryogenically cooled triple resonance probe (TCI) as previously described [1,4]. All NMR spectra were acquired using a 1D NOESY pulse sequence, with 80 Hz water suppression, 2 sec D1 delay, a sweep width of 12 ppm, and a 4 sec acquisition time. All spectra were processed using the TopSpin software package version 3.5 pl.7 (Bruker Biospin, Rheinstetten, Germany). The Chenomx NMR Suite software version 8.3 (Chenomx, Inc., Alberta, Canada) was used to identify and quantify the metabolites in the urine samples using the existing Chenomx spectral library, acquired with the identical NMR pulse sequence used for the urine samples. Quantification of urinary metabolites by NMR is not affected by interference from the protein/lipoprotein components that are common with serum and plasma. Ultrafiltration to remove these components was not required. Each metabolite concentration was corrected for dilution with the 5 × NMR buffer by dividing by 0.8. The µM/mM creatinine was calculated by dividing the µM of metabolite by the µM creatinine and then multiplying by 1000 µM/mM.

**RESULTS (SUPPLEMENTARY)**

***Initial optimization of the assay protocol using pyruvic acid***

As the DNPH reacts with any α-keto acid or ketone, we optimized our protocol using a simple, inexpensive α-keto acid, pyruvic acid. Pure, commercially available pyruvic acid reacted with DNPH and NaOH to produce a colored product detectable at 416 nm [5]. However, this reaction was not previously reported with urine. We demonstrated that pyruvic acid, a low abundance urinary metabolite (normal range 7-32 µM/mM creatinine [6,7]), when spiked into a colorless urine sample and reacted with DNPH, generated a pale-yellow solution with yellowish precipitates. These precipitates could be dissolved, producing clear, reddish-amber products immediately after NaOH addition. This demonstrated that an α-keto acid could be detected in urine using DNPH and NaOH. With increasing concentrations of pyruvic acid, a distinct color gradient was observed, suggesting that a simple color chart could be produced for facile analyte quantification. Two minutes after NaOH addition, we found two absorbance maxima (λ_max_) of the carboxylate products, one at 430 nm with a second minor λ_max_ at 520 nm (**Figure S1**) after scanning from 300 - 700 nm. The λ_max_ at 430 nm is slightly different from the reported 416 nm [5] (in pure water) likely due to metabolite in the urine leading to a slight red shift. When spiked pyruvic acid concentrations were plotted against A_430_ and A_520_ of the carboxylate products, a linear relationship was observed suggesting that a linear standard curve could be generated (data not shown).

This optimized protocol was tested using different urine samples and yielded the same color formation, demonstrating that consistent soluble, colored products could be generated without precipitates in different urine backgrounds. However, when intensely yellow-colored urine samples were assayed, inconsistent results were observed. This suggested that either interfering factors were present that prevented full color development or that other α-keto acids/ketones in urine were contributing to the color formation. This indicated further optimization of the assay was needed.

***Assay optimization for MSUD α-keto acids***

Since our DNPH protocol worked with a simple α-keto acid (pyruvate), we wanted to ensure our assay worked with the higher concentrations of α-keto acids expected in MSUD urine [8] (see **Table S1**). Based on published data, total α-keto acids in MSUD urine samples should range from 900-1600 μM/mM creatinine compared to 0.19- 3.22 μM/mM creatinine in normal urine samples [7] or 9000-16,000 μM total α-keto acids when 10 mM creatinine (normal) is present. This range covers >2 log units and would be difficult to measure for a useful at-home assay. Generally, urinary α-keto acids ranging from 300 µM to 2000 µM are desirable and reflect BCAA plasma levels in a healthy range of 75-300 µM for leucine and 200-400 µM each for isoleucine and valine [9-11]. We spiked urine with 0-2000 µM pyruvate and reacted the sample with DNPH/NaOH. When only 10 μL of DNPH solution was added, the intensely reddish-amber colored products were not observed. We then added increasing amounts of the DNPH (15, 20, and 30 μL), with 30 μL yielding the best color gradient. For all subsequent assays when 50 μL of urine was tested, 30 μL of the DNPH solution was added followed by 50 μL of 6 M NaOH. Note: some precipitates formed when the highest concentration (2000 μM) of pyruvate was reacted.

Since our goal was to develop a simple, at-home assay for MSUD, we used simple dilution to minimize the possible effects of interfering compounds and to avoid precipitation of the hydrazone compounds in patient samples containing high amounts of α-keto acids. With urine diluted 2X with water, significant sample-to-sample variation was observed (data not shown). With 3X diluted urine, consistent colors were observed, and no precipitation was seen, even with 1800 μM total α-keto acids. Based on these results, urine samples were diluted 3X before testing for the MSUD α-keto acids in our modified DNPH protocol.

After optimizing on pyruvic acid, we then demonstrated that the assay worked with a single α-keto acid, KIC (500-4000 µM), spiked into 3X diluted L-PU. With DNPH added, a yellow reaction mixture containing increasing amounts of yellowish-white precipitate was observed (**Figure S2a**). When NaOH was then added, the yellowish-white precipitates disappeared and an amber, reddish-amber to dark amber product was seen (**Figure S2b**), demonstrating that the assay works well with an α-keto acid found in urine of MSUD patients.

***Performance of the assay using MSUD α-keto acids spiked urine containing pyruvate and α-ketoglutarate***

While three α-keto acids (KIC, KIV, KMV) accumulate to high levels in the urine of MSUD patients, other less abundant and “normal” α-keto acids such as pyruvate, AKG, and ketones, such as acetone and acetoacetate, are also present in urine. Note that pyruvate (normal value: 7-32 µM/mM creatinine [7,12]) and AKG (normal value: 4-52 µM/mM creatinine [7,12]) have a combined “maximum α-keto acid concentration” of ~ 90 µM/mM creatinine in normal urine, or about 900 µM when 10 mM creatinine is present. To see if such a potentially high background of α-keto acids would adversely affect the color formation or linearity of our assay, we spiked our 3X diluted normal urine sample with 100 µM pyruvate and 200 µM AKG (equivalent to 900 µM total background α-keto acids) before adding increasing amounts of the MSUD α-keto acids KIV, KMV and KIC (see **Table S2** for composition of these keto acids). These spiked urine samples were then assayed using our DNPH protocol. As seen in **Figure S3a**, the unspiked, diluted urine sample produced little or no color and represents a typical background reaction for normal urine samples and can be set as a threshold that distinguishes healthy from unwell individuals. The remaining wells contained increasing amounts of MSUD α-keto acids with 300 µM pyruvate plus AKG. The amber-brown color increased in intensity with increasing amounts of α-keto acids (**Figure S3a**). After scanning from 300-700 nm, we observed absorbance maxima at 380 nm for KIC, KMV and AKG, at 430 nm for KIC and a second maximum at 530 nm for KIC, KIV, KMV and AKG (**Figure S1**). We chose the absorbance values at A_430_ and A_530_ where the absorbances were maximal_._ When these absorbances were plotted against the theoretical spiked concentrations, a linear calibration curve was generated for each wavelength with almost identical R^2^ (0.99; **Figure S3b**). This test demonstrated that our modified DNPH assay can quantitatively measure 300-1800 µM total α-keto acids in 3X diluted urine. Furthermore, it showed that the test would not confuse even extreme “normal” samples (containing high-normal levels of common α-keto acids such as pyruvate and AKG) with MSUD or with an MSUD “α-keto acid flare-up”.

**Table S1** Stability of study reagents and urine samples and number of replicates used in assays and inter-assay variability

| **Reagents or Urine Samples** | **Storage** | **Stability** |
| --- | --- | --- |
| 10 mM stock solutions of pyruvic acid, AKG*, KIC*, KIV*, KMV*, and PPA* | 100 µL aliquots, -20 ºC | 1 year |
| 4.4 mM dinitrophenylhydrazine (DNPH) (8.8 mg DNPH/10 mL 2 M hydrochloric acid) | -20 ºC | 1 year |
| 10 mM DNPH in 5 mL reaction vials (198 mg DNPH/100 mL 2M HCl) | Room temperature (RT) or 4 ºC, foil wrapped | 1 week RT or 12+ weeks 4 ºC |
| 6M NaOH | RT | indefinitely |
| NaOH immobilized with caprolactam | RT, covered with paraffin, stored in desiccator | 1 year |
| 10 Volunteer Urine Samples | 1 mL aliquots -20 ºC | 1 year |
| PKU Urine Samples (Before Breakfast, After Breakfast, After Lunch, After Dinner) | 1 mL aliquots, transferred to -80 ºC for long term storage | 1 year |
| Local Pooled Urine (L-PU) | 1 mL aliquots, -20 ºC | 1 year |
| Commercial Pooled Urine (C-PU) | 50 mL aliquots, -80 ºC | 5+ years |
| **Types of Samples** | **Replicates of Assays** | |
| MSUD or PKU calibrants, 96 well plate | n = 3 for each calibrant | |
| MSUD or PKU spiked volunteer samples (35), 96 well plate | n = 1 each variably spiked samples | |
| MSUD calibrants, at-home assay | n = 3 for each calibrant | |
| PKU calibrants, at-home assay | n = 3 for each calibrant | |
| PKU patient samples (4) | n = 3 each | |
| **Assay** | **Inter-assay Variability** | |
| At-home assay (OD_430_, spectrophotometry)^1^ | CV = 4.3% (n = 7) | |

*Abbreviations: AKG - α-ketoglutarate; KIC - α-ketoisocaproic acid; KIV - α-ketoisovaleric acid; KMC - α-keto-β-methylvaleric acid; MSUD - maple syrup urine disease; PKU -phenylketonuria; PPA - phenylpyruvic acid;

^1^CV (%) calculated from OD₄₃₀ values using the same aliquoted spiked urine sample and pre-prepared DNPH across independent assay runs conducted over a 12-week period (n = 7): (SD/mean) × 100.

**Table S2.** Composition of the keto acids mixtures tested using the DNPH-NaOH assay and expected ranges in normal and MSUD or PKU patients.

| **Keto acid alone or in mixtures** | **Final concentrations of keto acids (μM)** | **Composition of mixtures of keto acids** | **Expected ranges in normal or in-born error in metabolism (IEM) urine (µM)**  **when 10 mM creatinine present** | | |
| --- | --- | --- | --- | --- | --- |
| PYR spiked into neat urine | 0, 100, 200, 300, 400 | **0 μM** (endogenous keto acids)  Remainder-only PYR in all mixtures | 70-320 | | |
| KIC, KIV, KMV spiked into 1/3 diluted urine | 0  300  500  800  1000  1200  1600 | 0 μM (endogenous keto acids)  **300 µM** (100 μM each KIV, KMV, KIC)  **500 µM** (300 μM KIV, 100 μM each KMV, KIC)  **800 µM** (400 μM KIV, 200 μM each KMV, KIC)  **1000 µM** (500 μM KIV, 300 μM KMV, 200 μM KIC)  **1200 µM** (600 μM KIV, 400 μM KMV, 200 μM KIC)  **1600 µM** (800 μM KIV, 500 μM KMV, 300 μM KIC) | **Normal**  KIC 0-7  KIV 0.7-11  KMV 1.2-14.2 | **MSUD**  KIC 3680 ± 1160  KIV 3000-8000  KMV 2090 ± 350 (S); 660 ± 110 (R)  Total: ~9000-16,000 | |
| KIC, KIV, KMV with PYR and AKG spiked into 1/3 diluted urine | 0  300  600  800  1100  1300  1800 | **0 μM (**endogenous keto acids)  **300 µM** (100 μM PYR 200 μM AKG)  **600 µM** (100 μM PYR, 200 μM AKG, 100 μM KIV, 100 μM KMV, 100 μM KIC)  **800 µM** (100 μM PYR, 200 μM AKG, 300 μM KIV, 100 μM KMV, 100 μM KIC)  **1100 µM** (100 μM PYR, 200 μM AKG, 400 μM KIV, 300 μM KMV, 100 μM KIC)  **1300 µM** (100 μM PYR, 200 μM AKG, 500 μM KIV, 300 μM KMV, 200 μM KIC)  **1800 μM** (100 μM PYR, 200 μM AKG, 800 μM KIV, 500 μM KMV, 200 μM KIC) |  | | |
| PPA spiked into ¼ diluted urine | 0, 300, 500, 800, 1000, 1200, 1600 | **0 μM** (endogenous keto acids)  Remainder-only PPA in all mixtures | **Normal**  **Newborn** 0.0064 +/- 0.005  **1–13-year-old:** 2.5 +/- 4.3 | | **PKU**  **Newborn** 1466 +/- 2446  **1–13-year-old:** 8829.5 +/- 7410.7 |
| PPA with PYR and AKG spiked into ¼ diluted urine | 0  300  600  800  1100  1300  1800 | **0 μM** (endogenous keto acids)  **300 µM** (100 μM PYR and 200 μM AKG)  **600 µM** (300 µM PPA, 100 μM PYR, 200 μM AKG)  **800 µM** (500 µM PPA, 100 μM PYR, 200 μM AKG)  **1100 µM** (800 µM PPA, 100 μM PYR, 200 μM AKG)  **1300 µM** (1000 µM PPA, 100 μM PYR, 200 μM AKG)  **1800 µM** (1500 µM PPA, 100 μM PYR, 200 μM AKG) |  | | |

Abbreviations: AKG-α-ketoglutarate; KIC-α-ketoisocaproic acid; KIV- α-ketoisovaleric acid; KMC- α-keto-β-methylvaleric acid; MSUD -maple syrup urine disease; PPA – phenylpyruvic acid; PYR-pyruvate; PKU-phenylketonuria

**Table S3.** Standard deviation and coefficient of variation (%) for MSUD keto acids assay.

| **Absorbance, standard deviation (SD) and coefficient of variation (CV) at 430 nm or 530 nm** | **Concentration of keto acids (μM)** | | | | | |
| --- | --- | --- | --- | --- | --- | --- |
|  | 300 | 500 | 800 | 1000 | 1200 | 1600 |
| Average Absorbance at 430 nm | 0.23 | 0.39 | 0.62 | 0.75 | 0.88 | 1.08 |
| Average Absorbance at 530 nm | 0.14 | 0.23 | 0.35 | 0.44 | 0.50 | 0.61 |
| SD at 430 nm | 0.01 | 0.01 | 0.04 | 0.00 | 0.03 | 0.02 |
| CV (%) at 430 nm | 5.60 | 2.75 | 6.76 | 0.55 | 3.68 | 1.46 |
| SD at 530 nm | 0.01 | 0.01 | 0.02 | 0.01 | 0.02 | 0.01 |
| CV (%) at 530 nm | 5.75 | 2.95 | 6.04 | 2.10 | 3.55 | 1.98 |

**Table S4.** Standard deviation and coefficient of variation (%) for PKU phenylpyruvate assay.

| **Absorbance, standard deviation (SD) and coefficient of variation (CV) at 430 nm or 530 nm** | **Concentration of keto acids (μM)** | | | | | |
| --- | --- | --- | --- | --- | --- | --- |
|  | 300 | 500 | 800 | 1000 | 1200 | 1600 |
| Average Absorbance at 430 nm | 0.25 | 0.42 | 0.66 | 0.80 | 0.91 | 1.11 |
| Average Absorbance at 530 nm | 0.16 | 0.27 | 0.42 | 0.51 | 0.58 | 0.70 |
| SD at 430 nm | 0.01 | 0.02 | 0.04 | 0.05 | 0.05 | 0.02 |
| CV (%) at 430 nm | 4.19 | 5.42 | 5.58 | 5.86 | 5.59 | 1.74 |
| SD at 530 nm | 0.00 | 0.01 | 0.01 | 0.03 | 0.03 | 0.02 |
| CV (%) at 530 nm | 2.49 | 4.84 | 3.15 | 5.63 | 5.45 | 2.47 |

**Table S5.** Metabolites that react with DNPH to produce color and their percentage of absorbance relative to phenylpyruvic acid (PPA).

| **Interfering metabolites**  **(500 µM)** | **Absorbance** | **Net absorbance (background of unspiked urine subtracted from each)** | **Percentage of PPA net absorbance*** |
| --- | --- | --- | --- |
| AKG | 0.13 | 0.041 | 42.3 |
| PPA | 0.186 | 0.097 | 100.0 |
| 4-HPPA | 0.18 | 0.091 | 93.8 |
| Oxalacetate | 0.16 | 0.071 | 73.2 |
| Phenylacetate | 0.137 | 0.048 | 49.5 |
| Pyruvate | 0.166 | 0.077 | 79.4 |
| Unspiked | 0.089 | 0 | 0.0 |

*Percentage of PPA absorbance was calculated by dividing the net absorbance of the metabolite by the net absorbance of PPA and multiplying by 100 (see equation 2 in the main text).

Abbreviations: AKG – α-ketoglutaric acid; 4-HPPA - 4-hydroxyphenylpyruvic acid; PPA- phenylpyruvic acid


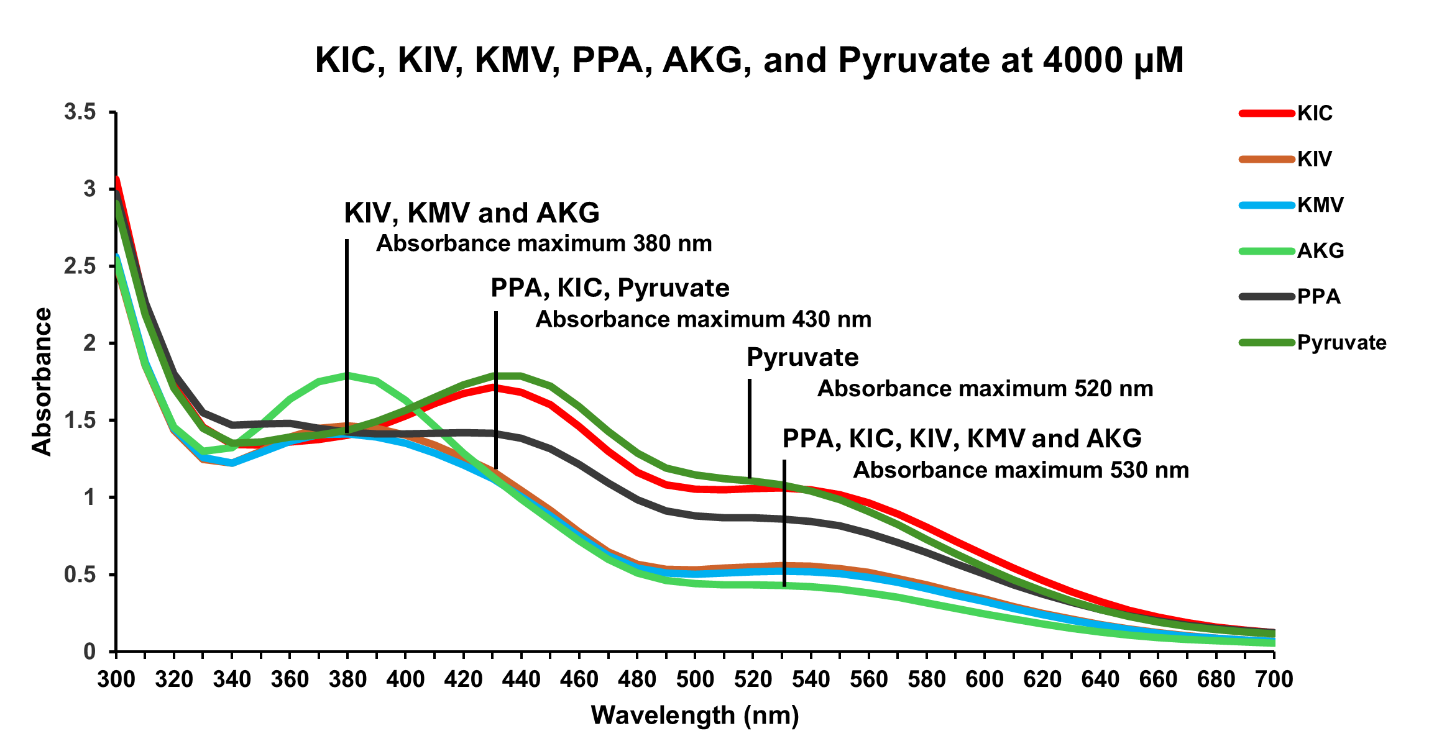


**Figure S1. Absorbance maxima of keto acids reacted with DNPH and NaOH.** Four concentrations (0, 1000, 2000, 4000 µM) of each keto acid (AKG – α-ketoglutarate, KIC - α-ketoisocaproic acid, KIV - α-ketoisovaleric acid, KMV - α-keto-β-methylvaleric acid, PPA – phenylpyruvate, and pyruvate) were spiked into commercial PU (C-PU) and reacted using the assay kit vials and dried NaOH caps. The colored products were transferred into a 96-well plate and scanned from 300 – 700 nm. Absorbance maxima from the 4000 µM spiked samples are shown as they were discernible at this concentration. PPA, KIC and pyruvate have a different absorbance maximum (430 nm) than KIV, KMV and AKG (380 nm). The second absorbance maximum also varies for pyruvate (520 nm) compared to the others (PPA, KIC, KIV, KMV and AKG at 530 nm).


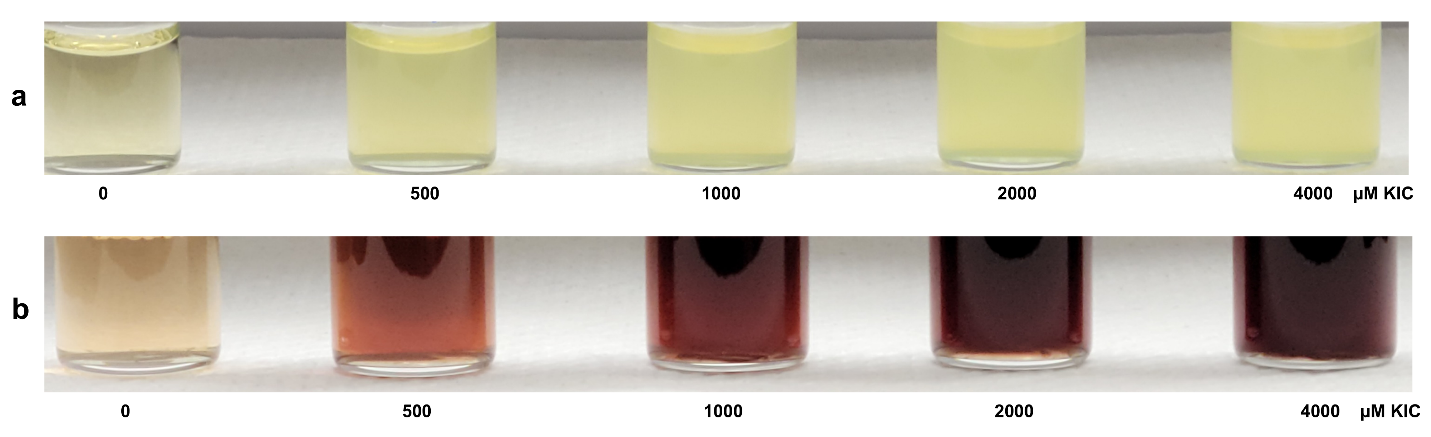


**Figure S2. Colored products after reaction of diluted local pooled urine (L-PU) with DNPH (a) followed by addition of a base (b).** (a) Yellow reaction mixture with precipitate after addition of 2, 4-DNPH to 3X diluted L-PU spiked with different concentrations of α-ketoisovaleric acid (KIC). (b) Clear amber-brown reaction mixture after the addition of NaOH.


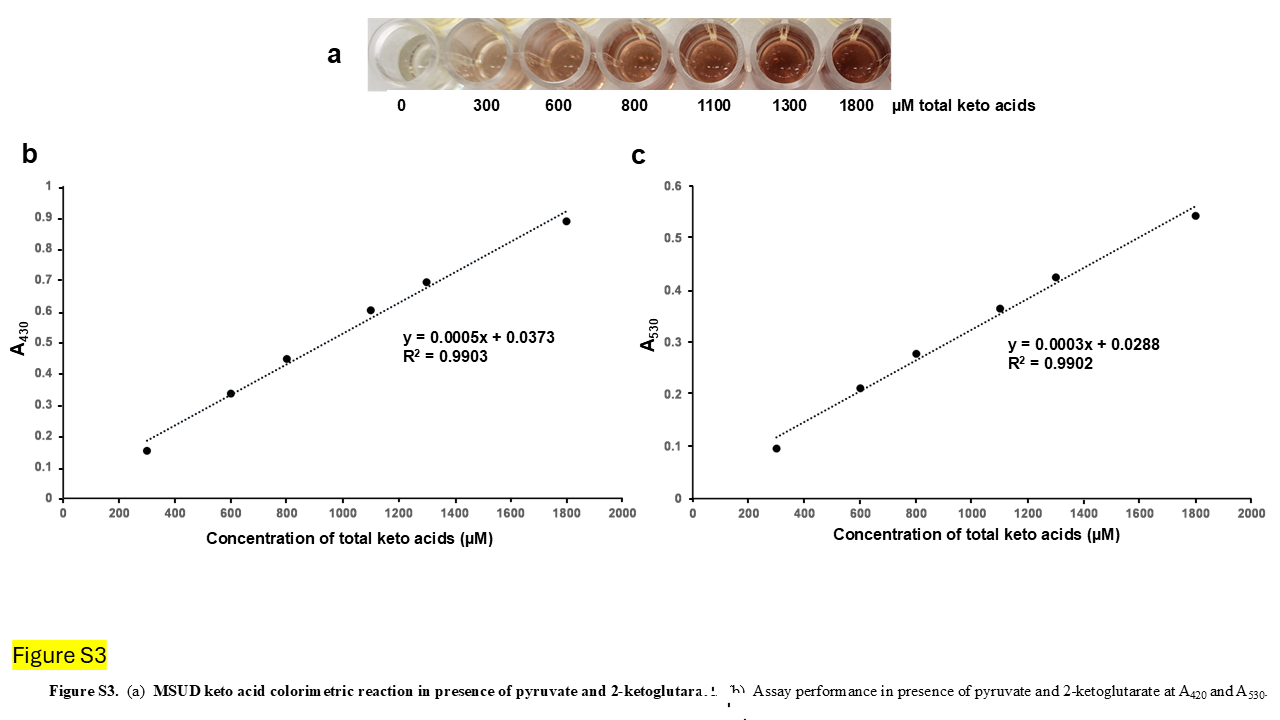


**Figure S3. (a) MSUD keto acid colorimetric reaction in presence of pyruvate and α-ketoglutarate (AKG).** (b) Assay performance in presence of pyruvate and AKG at A_430_ and A_530_.


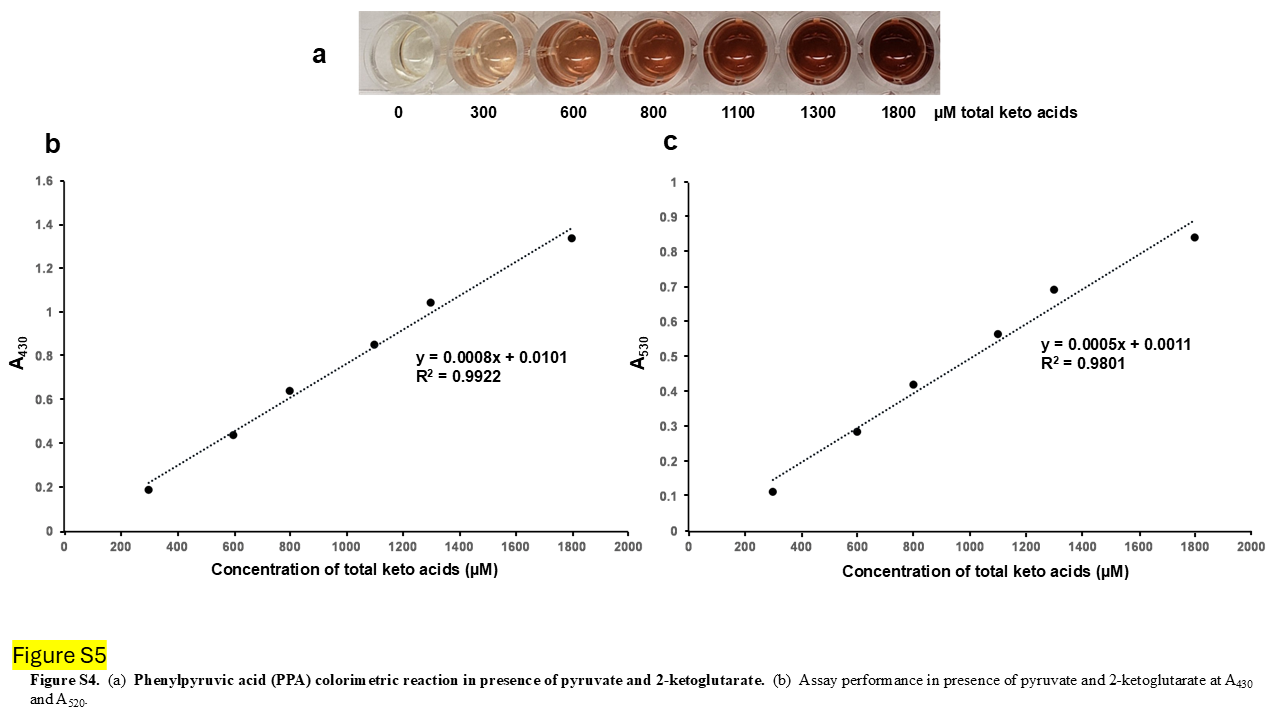


**Figure S4. (a) Phenylpyruvic acid (PPA) colorimetric reaction in presence of pyruvate and *α-*ketoglutarate (AKG).** (b) Assay performance in presence of pyruvate and AKG at A_430_ and A_530._


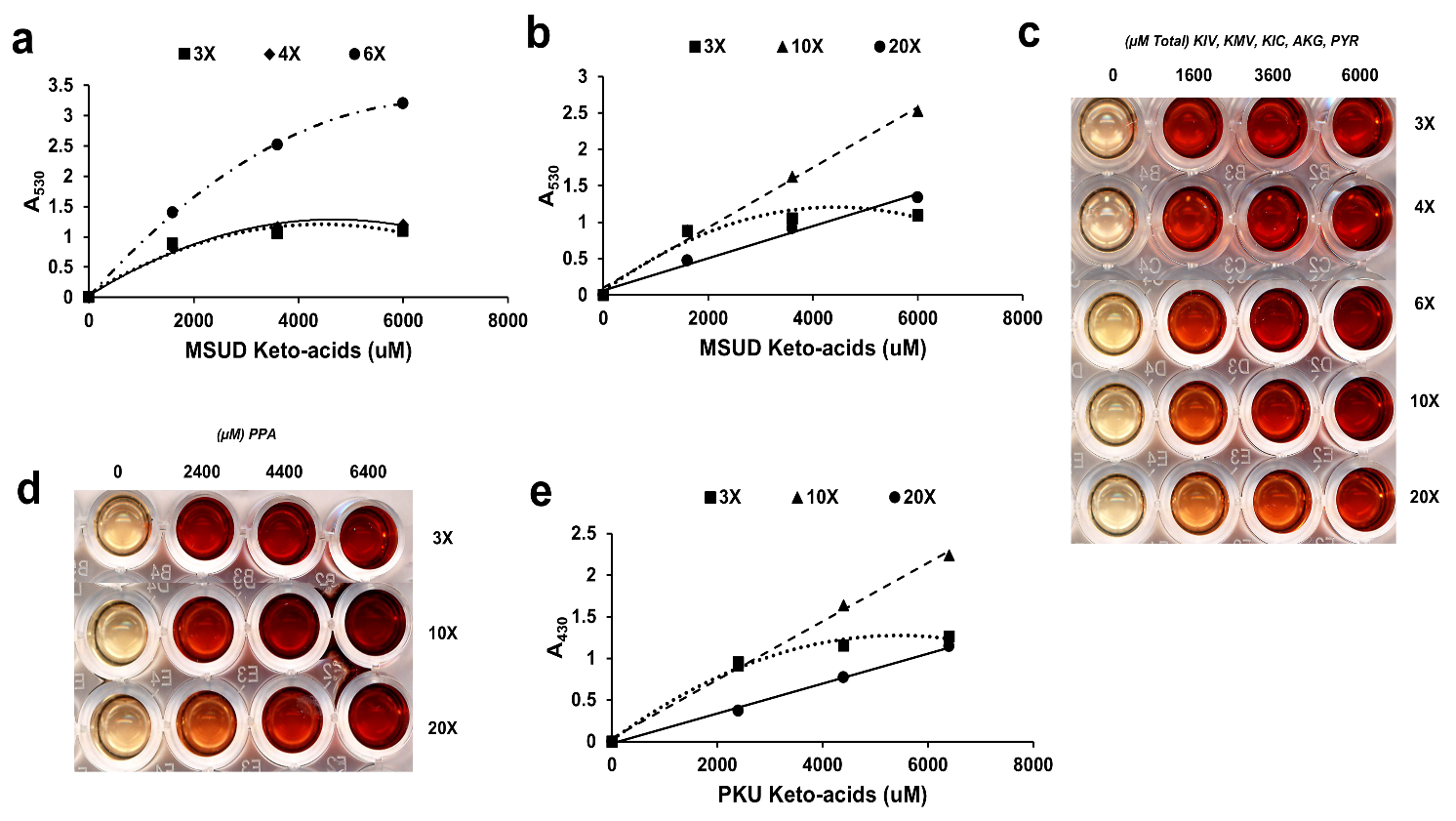


**Figure S5. Optimal dilution of urine samples needed to generate visually discernible colors and a linear response using the at-home assay**. MSUD (a, b, c) or PKU (d, e) keto acid spiked urine samples were diluted 3X, 4X, 6X, 10X and 20X with Milli-Q water and reacted via the at-home assay and transferred to 96-well plates. The 3X and 4X dilutions used 4.44 mM DNPH to minimize oversaturation at lower dilutions, while the 6X, 10X, and 20X dilutions used 10 mM DNPH. The 3X, 4X, and 6X dilutions were tested with the MSUD and PKU assays (data not shown), resulting in a non-linear response (a). The results for 3X, 10X, and 20X dilutions (b - MSUD, e - PKU) are presented for both assays, with 10X and 20X showing a linear response. The 20X dilution provided the clearest gradient with the most distinction between colors, as seen in the bottom row of the 96-well plates (c - MSUD, d - PKU).


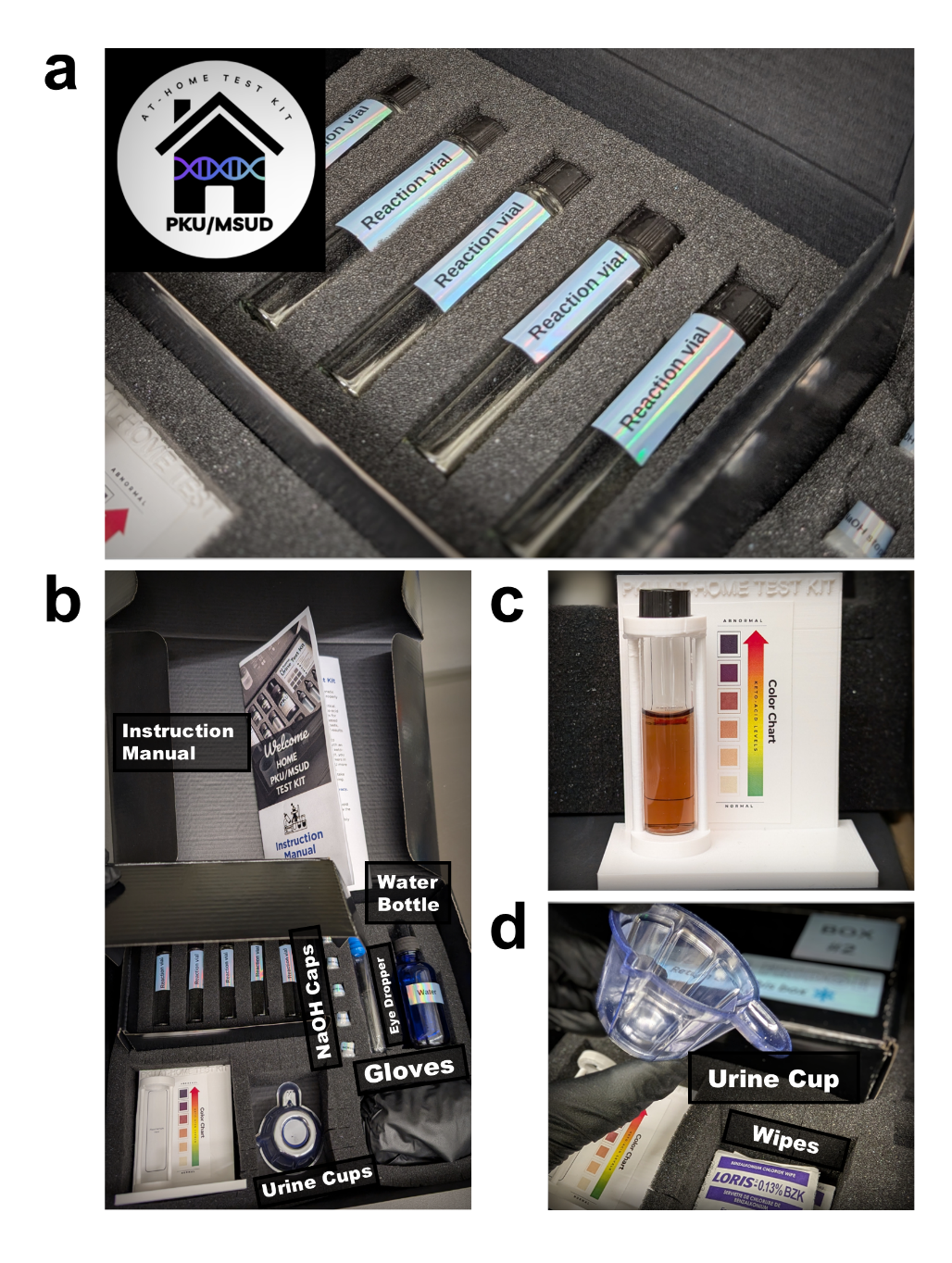


**Figure S6. PKU/MSUD Test Kit Prototype.** The kit contains dinitrophenylhydrazine (DNPH) reaction vials (a) marked with one line for the 20X water dilution, an instruction manual (b), NaOH stopper caps (b), a water dropper bottle for the fill-to-line dilution method (b), gloves (b), a 3D-printed vial stand with a printed sticker color chart for reference to keto acid levels (c), urine cups (d), and benzalkonium chloride (BZK) antiseptic wipes (d).


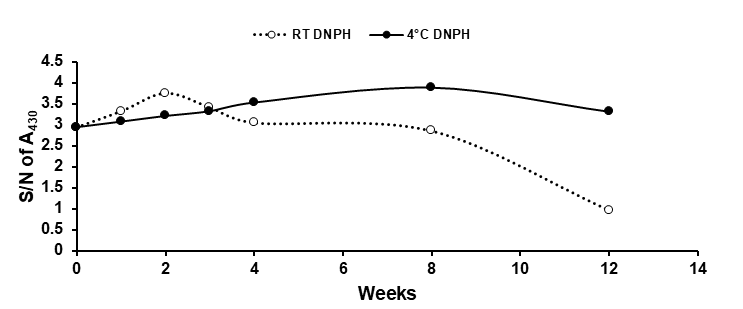


**Figure S7. Stability testing of the pre-measured dinitrophenylhydrazine (DNPH) stored in the kit vial and dried NaOH stoppers/caps.** DNPH reaction vials were stored at 4 °C and room temperature (RT) for up to 12 weeks. Spiked urine samples, 0 µM and 4000 µM phenylpyruvic acid - PPA, were aliquoted and stored at -80 ᣞC until testing to ensure stability of all metabolites over the 12 weeks of testing. At the indicated time points, DNPH vials (stored at RT or 4 ᣞC) and spiked urine samples (stored at -80 ᣞC) were removed, thawed and reacted via the at-home assay. Absorbance at 430 nm (A_430_) was measured and spectrophotometric readings over 12 weeks were assessed as signal-to-noise (S/N) ratios under both conditions for DNPH storage. Precipitates were seen with DNPH vials stored at RT while consistent readings were observed with the DNPH vials stored at 4 ᣞC.


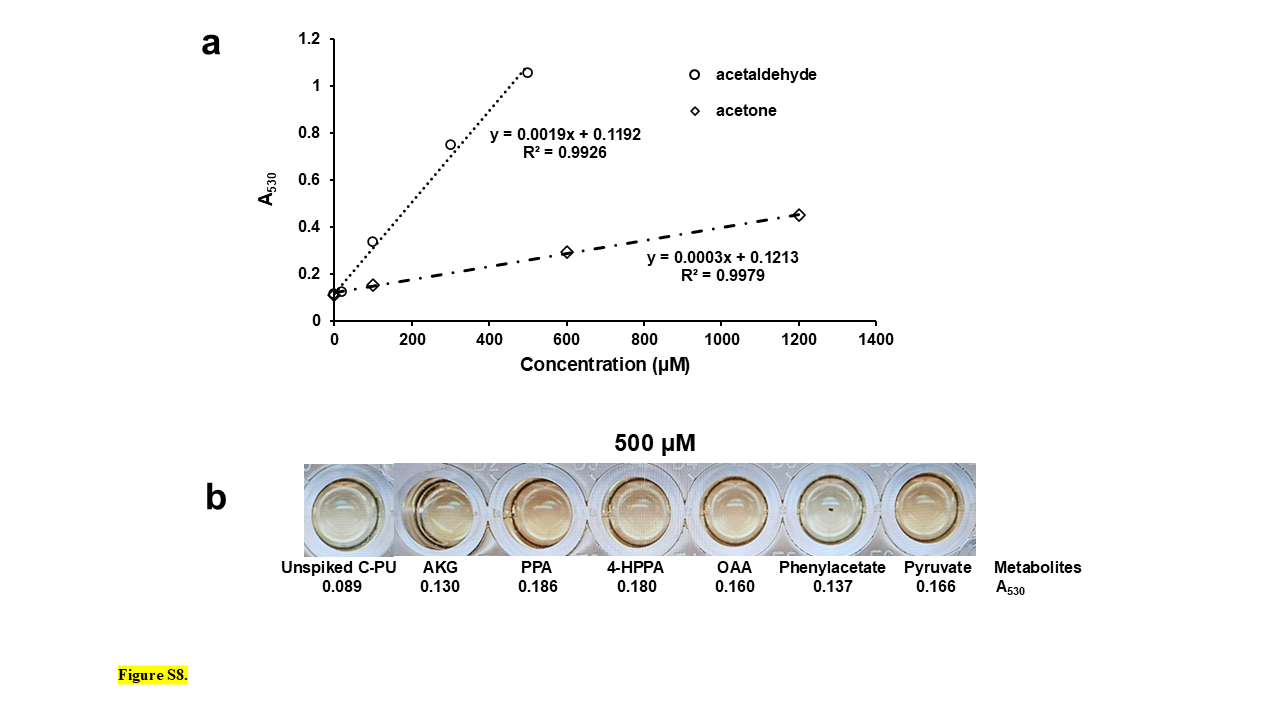


**Figure S8.** **Metabolites that react with DNPH and NaOH and contribute to the color of the at-home assay**. a) Acetone and acetaldehyde, prepared in water, were reacted via the at-home assay, with the resulting-colored products absorbances measured at 530 nm. With increasing amounts of acetaldehyde compared to acetone, greater absorbance was observed. b) Photographs of 500 µM of AKG, PPA, 4-HPPA, OAA, phenylacetate and pyruvate, spiked into commercial pooled urine (C-PU) and assayed via the at-home assay. Colored products were seen with all compounds except for unspiked C-PU and phenylacetate. Absorbances at 530 nm were measured when the products were transferred to 96 well plates. Except for the unspiked C-PU, increased absorbances were seen for all compounds including the colorless phenylacetate. Abbreviations: AKG – α-ketoglutaric acid; 4-HPPA - 4-hydroxyphenylpyruvic acid; OAA – oxalacetic acid; PPA- phenylpyruvic acid.

**
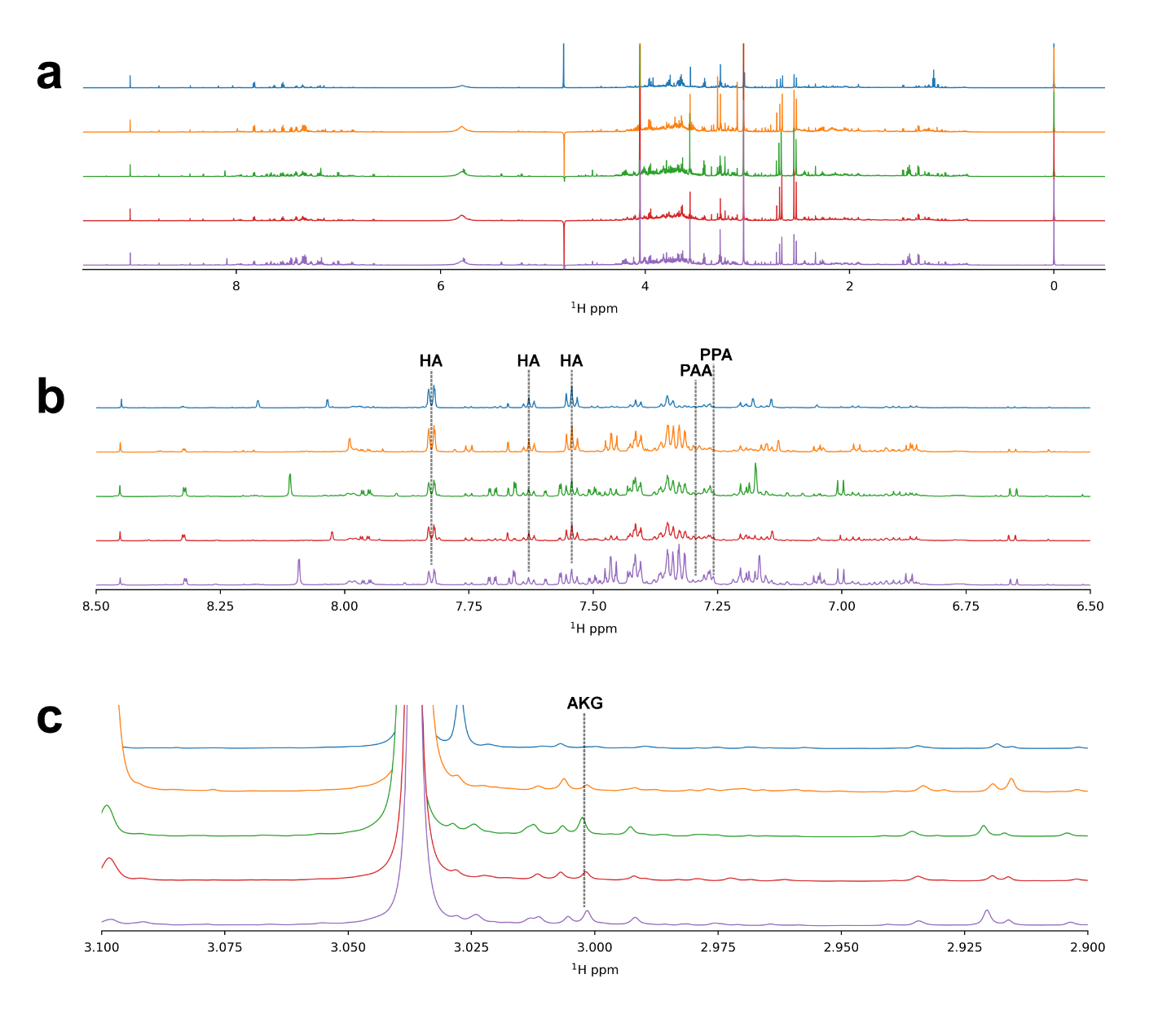
**

**Figure S9. ^1^H-Nuclear magnetic resonance (NMR) spectra from the commercial pooled urine (C-PU) and the four PKU urine samples, showing major peaks for α-ketoglutarate (AKG), phenylpyruvic acid (PPA), phenylacetic acid (PAA) and hippuric acid (HA).** In panels a-c, from top to bottom, are ^1^H-NMR spectra from C-PU (blue), PKU after an overnight fast (orange), PKU after breakfast (green), PKU after dinner (red) and PKU after lunch (purple). The order from top to bottom matches the left to right order of these samples in Table 2 of the manuscript. a) The full spectra from 0-10 ppm. b) The aromatic region (6.5 – 8.5 ppm) showing peaks for HA, PPA and PAA. c) Part of the aliphatic region (2.9 – 3.1 ppm) showing the position for AKG.

**REFERENCES**

1. D. Bhattacharyya, M. A. LeVatte, and D. S. Wishart, “A Fast and Accurate Colorimetric Assay for Quantifying Hippuric Acid in Human Urine,” *Analytical Biochemistry* 680 (2023): 115303, <https://doi.org/10.1016/J.AB.2023.115303>.

2. A. Shrivastava and V. Gupta, “Methods for the Determination of Limit of Detection and Limit of Quantitation of the Analytical Methods,” *Chronicles of Young Scientists* 2, no.1 (2011): 21, https://doi.org/10.4103/2229-5186.79345.

3. C. Cannet, A. Bayat, G. Frauendienst-Egger, et al., “Phenylketonuria (PKU) Urinary Metabolomic Phenotype Is Defined by Genotype and Metabolite Imbalance: Results in 51 Early Treated Patients Using Ex Vivo ^1^H-NMR Analysis,” *Molecules* 28, no.13 (2023): 4916, <https://doi.org/10.3390/molecules28134916>.

4. M. A. LeVatte, M. Lipfert, J. Zheng, et al., “A Fast, Sensitive, Single-Step Colorimetric Dipstick Assay for Quantifying Ascorbic Acid in Urine,” *Analytical Biochemistry* 580 (2019): 1–13, https://doi.org/10.1016/J.AB.2019.05.015.

5. H. Katsuki, T. Yoshida, C. Tanegashima, et al., “Improved Direct Method for Determination of Keto Acids by 2,4-Dinitrophenylhydrazine,” *Analytical Biochemistry* 43, no.2 (1971): 349–356, https://doi.org/10.1016/0003-2697(71)90263-6.

6. A. Koulman, P. Prentice, M. C. Y. Wong, et al., “The Development and Validation of a Fast and Robust Dried Blood Spot Based Lipid Profiling Method to Study Infant Metabolism,” *Metabolomics* 10, no.5 (2014): 1018–1025, https://doi.org/10.1007/s11306-014-0628-z.

7. D. S. Wishart, A. Guo, E. Oler, et al., “HMDB 5.0: The Human Metabolome Database for 2022,” *Nucleic Acids Research* 50, no.D1 (2022): D622–D631, https://doi.org/10.1093/nar/gkab1062.

8. P. Schadewaldt, H. W. Hammen, A. C. Ott, et al., “Renal Clearance of Branched-Chain L-Amino and 2-Oxo Acids in Maple Syrup Urine Disease,” *Journal of Inherited Metabolic Disease* 22, no.6 (1999): 706–722, https://doi.org/10.1023/A:1005540016376.

9. SA Hassan and V Gupta, *Maple Syrup Urine Disease*, 2014.

10. D. M. Frazier, C. Allgeier, C. Homer, et al., “Nutrition Management Guideline for Maple Syrup Urine Disease: An Evidence- and Consensus-Based Approach,” *Molecular Genetics and Metabolism* 112, no.3 (2014): 210–217, https://doi.org/10.1016/j.ymgme.2014.05.006.

11. B. Hoffmann, C. Helbling, P. Schadewaldt, et al., “Impact of Longitudinal Plasma Leucine Levels on the Intellectual Outcome in Patients with Classic MSUD,” *Pediatric Research* 59, no.1 (2006): 17–20, https://doi.org/10.1203/01.pdr.0000190571.60385.34.

12. “Lab Results Explained | HealthMatters.Io.” https://healthmatters.io/biomarker-category/metabolic-analysis-markers-urine . (accessed: 07-Jul-2021).
